# Comparison of Antiviral Agents for Seasonal Influenza Outcomes in Healthy Adults and Children: A Systematic Review and Network Meta-analysis

**DOI:** 10.1001/jamanetworkopen.2021.19151

**Published:** 2021-08-13

**Authors:** Jen-Wei Liu, Shen-Hua Lin, Lin-Chien Wang, Hsiao-Yean Chiu, Jen-Ai Lee

**Affiliations:** 1 Department of Pharmacy, Fu Jen Catholic University Hospital, Fu Jen Catholic University, New Taipei City, Taiwan; 2 School of Pharmacy, College of Pharmacy, Taipei Medical University, Taipei, Taiwan; 3 School of Nursing, College of Nursing, Taipei Medical University, Taipei, Taiwan; 4 Research Center of Sleep Medicine, College of Medicine, Taipei Medical University, Taipei, Taiwan

## Abstract

**Importance:**

Antiviral treatment of influenza is recommended for patients with influenza-like illness during periods of community cocirculation of influenza viruses and SARS-CoV-2; however, questions remain about which treatment is associated with the best outcomes and fewest adverse events.

**Objective:**

To compare the efficacy and safety of neuraminidase inhibitors and the endonuclease inhibitor for the treatment of seasonal influenza among healthy adults and children.

**Data Sources:**

Medline, Embase, and the Cochrane Register of Clinical Trials were searched from inception to January 2020 (the last search was updated in October 2020).

**Study Selection:**

Included studies were randomized clinical trials conducted among patients of all ages with influenza treated with neuraminidase inhibitors (ie, oseltamivir, peramivir, zanamivir, or laninamivir) or an endonuclease inhibitor (ie, baloxavir) compared with other active agents or placebo.

**Data Extraction and Synthesis:**

Two investigators identified studies and independently abstracted data. Frequentist network meta-analyses were performed; relative ranking of agents was conducted using P-score probabilities. Quality of evidence was assessed using the Grading of Recommendations, Assessment, Development and Evaluations criteria. Data were analyzed in October 2020.

**Main Outcomes and Measures:**

The time to alleviation of influenza symptoms (TTAS), complications of influenza, and adverse events (total adverse events, nausea, and vomiting).

**Results:**

A total of 26 trials were identified that investigated antiviral drugs at high or low doses; these trials included 11 897 participants, among whom 6294 (52.9%) were men and the mean (SD) age was 32.5 (16.9) years. Of all treatments comparing with placebo in efficacy outcomes, high-quality evidence indicated that zanamivir was associated with the shortest TTAS (hazard ratio, 0.67; 95% CI, 0.58-0.77), while baloxavir was associated with the lowest risk of influenza-related complications (risk ratio [RR], 0.51; 95% CI, 0.32-0.80) based on moderate-quality evidence. In safety outcomes, baloxavir was associated with the lowest risk of total adverse events (RR, 0.84; 95% CI, 0.74-0.96) compared with placebo based on moderate-quality evidence. There was no strong evidence of associations with risk of nausea or vomiting among all comparisons, except for 75 mg oseltamivir, which was associated with greater occurrence of nausea (RR, 1.82; 95% CI, 1.38-2.41) and vomiting (RR, 1.88; 95% CI, 1.47-2.41).

**Conclusions and Relevance:**

In this systematic review and network meta-analysis, all 4 antiviral agents assessed were associated with shortening TTAS; zanamivir was associated with the shortest TTAS, and baloxavir was associated with reduced rate of influenza-related complications.

## Key Points

**Question:**

What antiviral agents for treating seasonal influenza are associated with the most safety and best outcomes among healthy adults and children?

**Findings:**

This network meta-analysis of 26 randomized clinical trials, including 11 897 patients, found that antiviral agents were associated with significantly greater efficacy than placebo in shortening the duration of influenza symptoms; zanamivir was associated with the shortest time to alleviation of influenza symptoms. Baloxavir was associated with a lower occurrence of influenza-related complications than other treatments.

**Meaning:**

These findings suggest that zanamivir may be initiated as soon as possible for patients with influenza-like illness; in those who may be at high risk of developing influenza-related complications, baloxavir should be considered.

## Teaser

This systematic review and network meta-analysis assesses neuraminidase inhibitors and an endonuclease inhibitor for safety and efficacy against seasonal influenza in children and adults.

## Introduction

Seasonal influenza is a serious global health threat of high prevalence that affects all countries. During the SARS-CoV-2 pandemic, coinfection with influenza A or B viruses and SARS-CoV-2 can occur and should be considered. Influenza and COVID-19 have overlapping signs and symptoms.^1^ Studies have found that patients with COVID-19 who were coinfected with influenza shed SARS-CoV-2 longer than other patients with only COVID-19.^2^ Therefore, empirical antiviral treatment is recommended as soon as possible, particularly for hospitalized patients with severe respiratory disease, outpatients with influenza-like illness, and patients with higher risk for influenza complications.^1^

Antiviral medications can help to shorten the duration of illness and prevent serious complications. Neuraminidase inhibitors selectively block an enzyme conserved within all influenza viruses to inhibit virus particles from exiting host cells and thus interrupt person-to-person viral spread. Neuraminidase inhibitors also shorten the duration of symptoms, likely by decreasing the viral load, preventing the spread and release of cytokines, and reducing the risk of complications.^3^ Another novel oral antiviral drug, baloxavir marboxil (hereafter, *baloxavir*), is a selective inhibitor of influenza cap-dependent endonuclease, and blocks proliferation of the influenza virus by inhibiting the initiation of mRNA synthesis.^4^

The Centers for Disease Control and Prevention has recommended 4 Food and Drug Administration–approved antiviral drugs for the treatment of influenza: oseltamivir, baloxavir (oral), zanamivir (inhaled), and peramivir (intravenous).^5,6^ When indicated, treatment should be initiated as soon as possible within the first 48 hours of symptoms developing. The adverse effects of each medication vary but generally are gastrointestinal. The most common adverse effects of oseltamivir are nausea and vomiting; zanamivir may cause bronchospasm; and peramivir may cause diarrhea.^6^ However, owing to a lack of high-quality evidence, questions about which treatment is associated with the best outcomes and fewest adverse events remain. In this study, we aimed to compare the efficacy and safety outcomes associated with different antiviral agents for adults and children with seasonal influenza.

## Methods

This systematic review and network meta-analysis was performed in accordance with the PRISMA Extension Statement for Reporting of Systematic Reviews Incorporating Network Meta-analyses of Health Care Interventions (PRISMA-NMA). The study protocol was published on the PROSPERO international prospective register of systematic reviews (CRD42020186433).

### Eligibility Criteria

We included all published randomized clinical trials that compared the use of neuraminidase inhibitors (ie, oseltamivir, zanamivir, peramivir, and laninamivir) or endonuclease inhibitor (ie, baloxavir) with each other or with a placebo for the treatment of seasonal influenza. Studies assessing previously healthy people of all ages (children and adults) were included. Influenza-like illness was defined as a fever (≥38.0 °C), and at least 2 of the following 4 symptoms: headache, sore throat, myalgia, and cough, with a duration of symptoms of 48 hours or less.

### Outcomes and Summary Measures

The primary efficacy outcome was time to symptom alleviation (TTAS), defined as time from the start of treatment to patient-reported improvement in all influenza-associated symptoms and measured using hazard ratios (HRs). When expressing the effect size of an intervention for time-to-event data, HRs were the most appropriate. Using median or mean time-to-event for continuous outcomes may introduce bias, given that censored participants must be excluded.^7^ Many clinical trials only report treatment effects as median or mean values, which were insufficient for our analysis. We used DigitizeIt software version 2.2 (DigitizeIt) to reconstruct the Kaplan-Meier curves and obtain the HRs and 95% CIs.^8^ The reliability and validity of the data obtained using this tool were confirmed by comparing the results with those of other data extraction programs.^9^ Most studies reported Kaplan-Meier curves in laboratory-confirmed population. We made the decision about using the intention-to-treat-influenza-infected (ITTI) population to enable a more comprehensive comparison of the effectiveness of all included antiviral agents.

The secondary efficacy outcome was complications of influenza, also based on the ITTI population. Complications of particular interest included pneumonia, bronchitis, otitis media, sinusitis, and other secondary illness, whether treated with antibiotics or not.

The safety outcomes were total adverse events, nausea, and vomiting targeting the safety population. We chose nausea and vomiting as our safety end points because they were common, especially in young adults, and studies also showed that nausea and vomiting were associated with discontinuation of study drugs.^10,11^ Both outcomes of complications and adverse events were binary, reported as risk ratios (RRs) and 95% CIs. These outcomes were defined according to the authors of each study and were not exactly the same in all trials (eTable 1 in the Supplement).^10,11,12,13,14,15,16,17,18,19,20,21,22,23,24,25,26,27,28,29,30,31,32,33,34,35,36,37,38,39,40,41,42^

### Data Sources and Extraction

Comprehensive searches were conducted of Medline, Embase, and the Cochrane Register of Clinical Trials from inception until January 2020 (eAppendix 1 in the Supplement). We updated the last search in October 2020. The literature retrieval was independently carried out by 2 of us (J.-W.L. and S.-H.L.). There were no language restrictions. ClinicalTrials.gov was used to search clinical trial registers. We did not search for unpublished studies or contact experts in the field.

Two of us (J.-W.L. and S.-H.L.) independently extracted data from included trials, including published year, participants (mean age, inclusion criteria, influenza test results, and illness severity), interventions, results (outcome measures, TTAS, complications, total adverse events, nausea, and vomiting), and study design. Disagreements in extracted data were resolved by discussion.

### Risk of Bias and Quality of Evidence Assessment

We summarized the potential biases for each individual study using the Cochrane risk of bias tool.^43^ We also presented information related to potential issues of clinical heterogeneity for each study. For the network meta-analysis, the quality of direct and network evidence was graded using Grading of Recommendations Assessment, Development, and Evaluation.^44,45,46^ For each comparison, 2 of us (J.-W.L. and S.-H.L.) independently rated the quality of direct and network evidence for each outcome; discrepancies were resolved by consensus, and if necessary, with arbitration by a third author (J.-A. L.).

### Statistical Analysis

Network meta-analyses were fitted to a frequentist framework using a multivariate random-effects network meta-analyses model with the statistical package *netmeta* in R statistical software version 4.0.2 (R Project for Statistical Computing).^47^ For sparse binary outcome data (individual trials with zero events in either group), a continuity correction of 0.5 was added to each cell for each effect measure.^7,48^ We also pooled odds ratio outcomes using the Mantel-Haenszel method, which offers a reliable approach to assess binary outcomes, especially in the case of sparse data.^49^

We produced network plots for each outcome to visualize network geometry and node connectivity.^50^ We estimated the ranking probabilities of the different antiviral agents based on their P-scores. The P-score is measured on a scale from 0 (worst) to 1 (best), with a higher score indicating better overall performance of the competing treatment. The numerical P-score values are nearly identical to the surface under the cumulative ranking curve.^51^ It is also important to consider the relative risk and corresponding 95% CI for each comparison when interpreting the ranking results.^52^

We explored the transitivity assumption by assessing whether the distribution of patients and study characteristics, which could potentially modify the treatment effects across treatment comparisons, were significantly different.^7^ We assessed network heterogeneity across all treatment contrasts using τ^2^ and *I*^2^ statistics. We applied the *Q* statistic to test for global inconsistency using a design-by-treatment interaction random effects model.^53^ We evaluated local inconsistency via a node split method by splitting the network estimates into direct and indirect evidence using a back-calculation method.^54,55,56^ *P* values were 2-sided, and *P* values less than .05 were considered statistically significant.

We performed sensitivity analyses for the network meta-analysis outcomes of TTAS, nausea, and vomiting (self-report outcomes) to assess the robustness of the model by excluding trials with a high risk of bias in all domains overall and in the domain of blinding. We explored the potential for publication bias by visual inspection of the comparison-adjusted funnel plots and Egger test.^7,46,57^ We hypothesized that studies that find a new form of treatment is superior to an existing treatment may have a higher chance of getting published. Symmetry around the point estimate line in the scatter plot may indicate the absence of publication bias, or small study effects. Data were analyzed in October 2020.

## Results

### Study Selection and Characteristics

**Figure 1.**
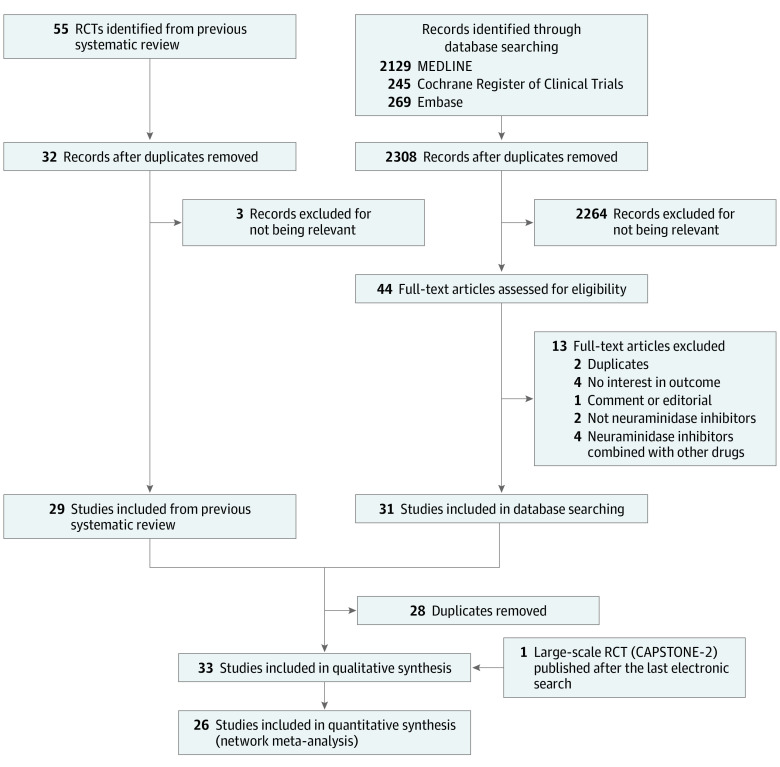
Flowchart of Study Selection RCT indicates randomized clinical trial.

As shown in Figure 1, we identified 33 potentially eligible, completed randomized clinical trials,^10,11,12,13,14,15,16,17,18,19,20,21,22,23,24,25,26,27,28,29,30,31,32,33,34,35,36,37,38,39,40,41,42^ after excluding 13 studies.^58,59,60,61,62,63,64,65,66,67,68,69,70^ Two studies (6%) published in Chinese^12,13^ and 1 study (3%) in Japanese^20^ were translated and included. A large-scale randomized clinical trial (CAPSTONE-2),^11^ published after our last electronic search, was also included.

Participants included children and adults of all ages, with 12 trials^15,21,22,23,24,28,29,31,33,35,37,42^ (36%) assessing adult populations (age ≥18 years) and 5 trials^17,18,34,38,41^ (15%) including only children. Most trials recruited patients attending outpatient clinics (29 trials^10,11,12,13,14,15,16,17,18,20,21,22,23,24,25,26,27,28,29,30,32,33,34,35,36,38,40,41,42^ [88%]); 4 trials (12%) included hospitalized patients with rapid antigen test (RAT) results positive for influenza.^19,31,37,39^

We focused on 4 Food and Drug Administration–approved antiviral agents at high or low doses: 3 neuraminidase inhibitors (75 mg or 150 mg oseltamivir twice daily, 10 mg zanamivir twice daily, and 300 mg or 600 mg peramivir once daily or a single dose) and 1 endonuclease inhibitor (single 40 mg or 80 mg dose of baloxavir). Laninamivir was not included in this meta-analysis because no available primary efficacy outcome data were available, and the drug was only investigated in a few Japanese trials.^38,41,42^ Finally, 26 trials with 11 897 participants were included in the quantitative synthesis (network meta-analysis) (eTable 1 in the Supplement).^10,11,12,13,14,15,16,17,18,19,20,21,22,23,24,25,26,27,28,29,30,31,32,33,34,35,36,37,38,39,40,41,42^ Across the included studies, 6294 (52.9%) were men, and the mean (SD) age was 32.5 (16.9) years.

### Risk of Bias of Included Studies

The risk of bias judgments for the studies contributing to the analysis of each outcome are presented in eFigure 1 in the Supplement. Open-labeled trials were determined to be at high risk of bias in the domain of blinding. Overall, 2 studies^17,68^ (6.1%) were judged to have a low risk of bias across all domains.

### TTAS

**Figure 2.**
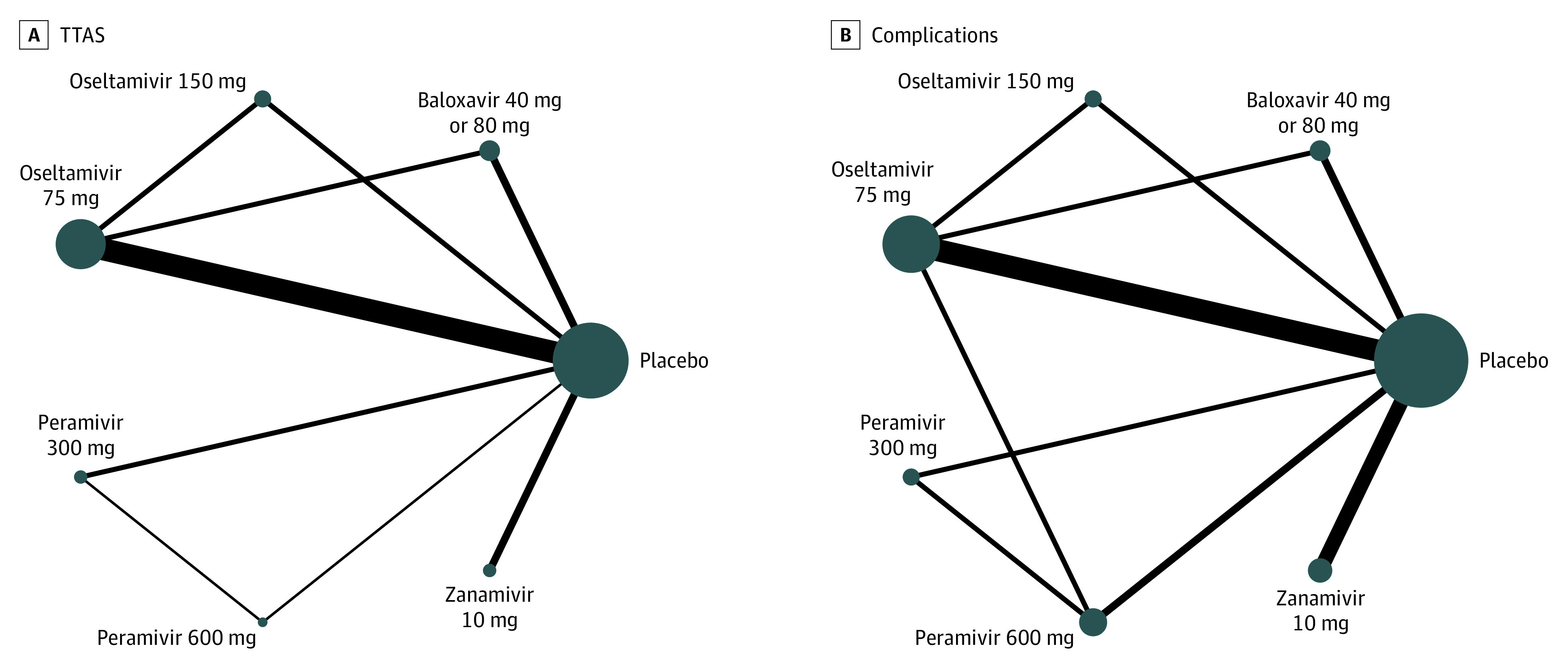
Network Graphs of Treatment Comparisons for Time to Alleviation of Influenza Symptoms and Complications Nodes indicate different active interventions or placebo; size of nodes, number of studies; thickness of lines between nodes, number of randomized participants contributing to direct comparisons.

**Figure 3.**
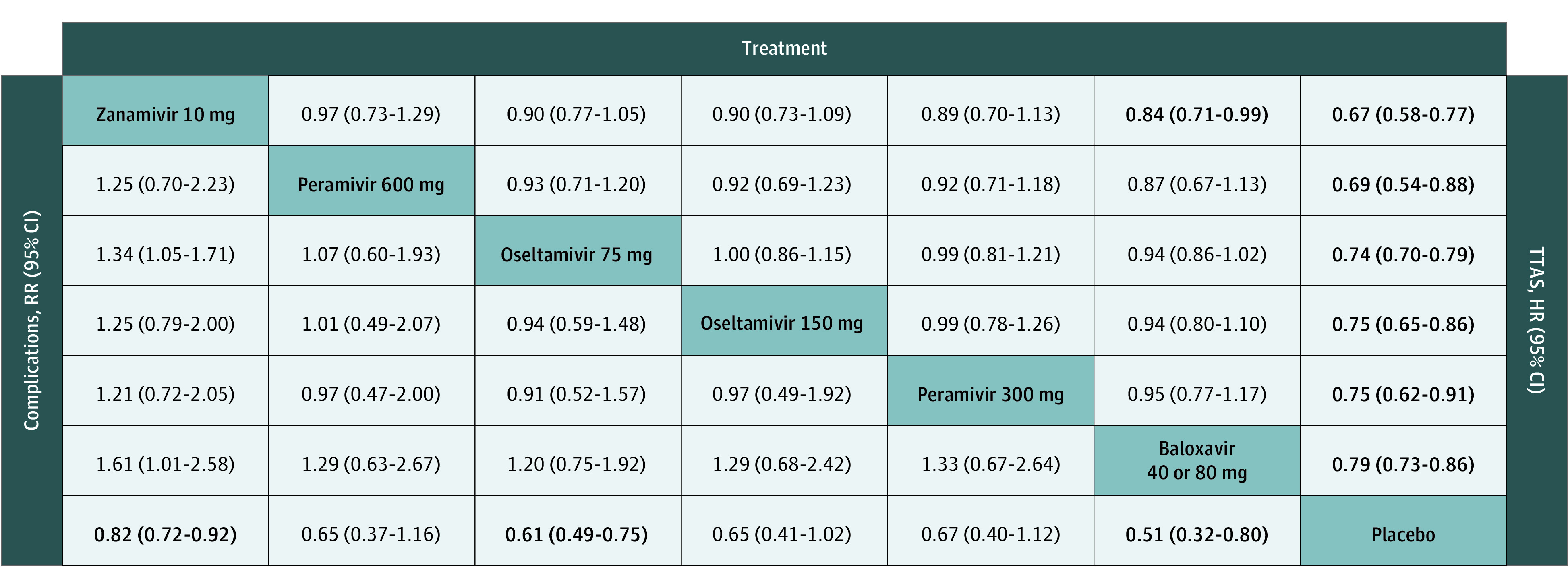
Network Meta-analysis Estimates of Time to Alleviation of Influenza Symptoms (TTAS) and Complications Effect sizes presented on the upper triangle correspond to hazard ratio (HR) (95% CI) of TTAS between the row and columns (eg, the HR of TTAS between zanamivir 10 mg and placebo is 0.97 [0.73-1.29]); On the lower triangle, effect sizes correspond to risk ratio (RR) (95% CI) of complications between the column and the row (eg, the RR [95% CI] of complications between zanamivir 10 mg and placebo is 0.82 [0.72-0.92]).

**Figure 4.**
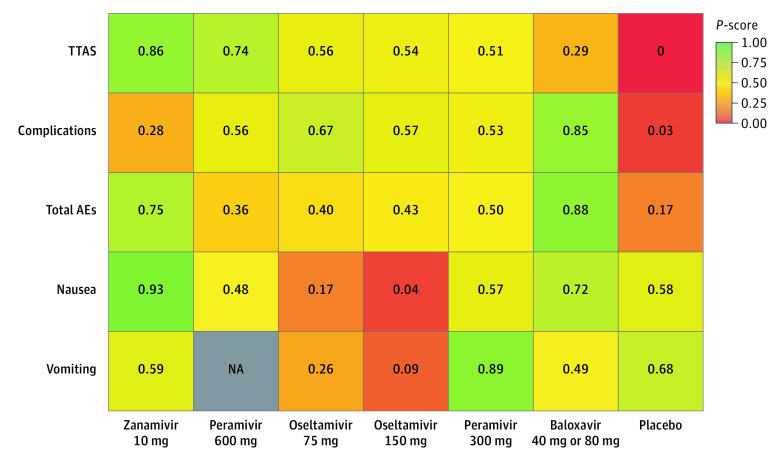
Heat Map of Antiviral Drugs Ranked According to Time to Alleviation of Influenza Symptoms (TTAS), Complications, Total Adverse Events (AEs), Nausea, and Vomiting Numbers reflect P-score, measured on a scale from 0 (worst) to 1 (best). A higher P-score indicates better effectiveness and fewer adverse events. Grey square indicates that data were not available (NA).

Among the ITTI population, the network of treatment comparisons for TTAS included 7 individual nodes among 15 trials (Figure 2A).^10,11,15,17,18,20,21,23,24,25,29,32,33,34,35^ No trial reported relevant TTAS data for laninamivir. Placebo was the most well-connected intervention and was directly linked to all other interventions. A total of 5 trials^10,11,21,29,32^ (33.3%) had 3 eligible groups, and the remaining 10 trials^15,17,18,20,23,24,25,33,34,35^ (66.7%) had 2 eligible groups. For the comparison of all treatments with placebo, 10 mg zanamivir was associated with the shortest TTAS (HR, 0.67; 95% CI, 0.58-0.77; P-score, 0.86), followed by 600 mg peramivir (HR, 0.69; 95% CI, 0.54-0.88; P-score. 0.74), 75 mg oseltamivir (HR, 0.74; 95% CI, 0.70-0.79; P-score, 0.56), 150 mg oseltamivir (HR, 0.75; 95% CI, 0.65-0.86; P-score, 0.54), 300 mg peramivir (HR, 0.75; 95% CI, 0.62-0.91; P-score, 0.51), and 40 mg or 80 mg baloxavir (HR, 0.79; 95% CI, 0.73-0.86; P-score, 0.29) (Figure 3 and Figure 4).

In the network meta-analysis, we found no evidence of heterogeneity (*τ*^2^ = 0 and *I*^2^ = 0%; 95% CI, 0%-50.2%). There was no measurable global inconsistency based on a random effects design-by-treatment model (χ^2^_4_ = 2.37; *P* = .67) (eTable 2 in the Supplement) or local inconsistency within the network (eAppendix 2 in the Supplement).

### Complications

Among the ITTI population, the network of treatment comparisons for complications reported from 21 trials^10,11,12,13,15,17,19,21,22,23,24,25,26,27,28,29,30,32,33,34,35^ was constituted of 7 individual nodes (Figure 2B). The placebo was the most well-connected group and was directly linked to all other interventions. In the comparison of all treatments with placebo, 40 mg or 80 mg baloxavir was associated with fewer influenza-related complications (RR, 0.51; 95% CI, 0.32-0.80; P-score, 0.85), followed by 75 mg oseltamivir (RR, 0.61; 95% CI, 0.49-0.75; P-score, 0.67), 150 mg oseltamivir (RR, 0.65; 95% CI, 0.41-1.02; P-score, 0.57), 600 mg peramivir (RR. 0.65; 95% CI, 0.37-1.16; P-score, 0.56), 300 mg peramivir (RR, 0.67; 95% CI, 0.40-1.12; P-score, 0.53), and 10 mg zanamivir (RR, 0.82; 95% CI, 0.72-0.92; P-score, 0.28) (Figure 3 and Figure 4).

No heterogeneity was observed (τ^2^ = 0 and *I*^2^ = 0%; 95% CI, 0%-41.6%). There was no measurable global inconsistency based on a random effects design-by-treatment model (χ^2^_9_ = 5.05; *P* = .83) (eTable 2 in the Supplement) or local inconsistency within the network (eAppendix 2 in the Supplement).

### Safety Outcomes

We established pooled estimates for total adverse events, nausea, and vomiting among the as-treated populations. The network graphs are presented in eFigure 2 in the Supplement, and the league tables are presented in eFigure 3 in the Supplement. The ranking of the treatment comparisons are shown in a heatmap in Figure 4.

For total adverse events, 21 trials^10,11,12,13,14,15,16,17,19,20,21,22,23,24,25,26,28,30,31,33,35^ compared 7 different interventions (eFigure 2 in the Supplement). There were almost no differences among the multiple treatment comparisons; only 40 mg or 80 mg baloxavir was associated with significantly fewer adverse events than the placebo (RR, 0.84; 95% CI, 0.74-0.96; P-score, 0.85) (eFigure 3 in the Supplement). Ranking of the risk of any adverse events identified 40 mg or 80 mg baloxavir as the best, indicating fewer adverse events, and peramivir 600 mg as the worst among all antiviral agents (Figure 4). No heterogeneity was observed (τ^2^ = 0 and *I*^2^ = 0%; 95% CI, 0%-42.7%). There was no measurable global inconsistency based on a random effects design-by-treatment model (χ^2^_8_ = 6.42; *P* = .60) (eTable 2 in the Supplement) or local inconsistency within the network (eAppendix 2 in the Supplement).

For nausea, 15 trials^10,11,14,17,19,20,21,23,24,25,26,28,29,32,35^ compared 7 different interventions (eFigure 2 in the Supplement). Ranking identified 10 mg zanamivir as the best and 150 mg oseltamivir as the worst (Figure 4). No heterogeneity was observed (τ^2^ = 0 and *I*^2^ = 0%; 95% CI, 0%-44.6%). There was no measurable global inconsistency based on a random effects design-by-treatment model (χ^2^_5_ = 7.89; *P* = .16) (eTable 2 in the Supplement). We only identified 1 contrast of inconsistency, indicating disagreement between the indirect and direct evidence for 150 mg oseltamivir vs placebo (eAppendix 2 in the Supplement). We found that 75 mg oseltamivir was associated with higher occurence of nausea vs placebo (RR, 1.82; 95% CI,1.38-2.41) (eTable 3 in the Supplement). Compared with 75 mg oseltamivir, zanamivir (RR, 0.30; 95% CI, 0.13-0.67) and baloxavir (risk ratio 0.47; 95% CI, 0.30-0.72) were associated with a lower occurrence of nausea. (eFigure 3 in the Supplement).

For vomiting, 9 trials^10,14,17,18,20,23,29,32,35^ compared 6 different interventions (eFigure 2 in the Supplement). No trial reported relevant data for 600 mg peramivir. In the ranking, 300 mg peramivir was identified as the best and 150 mg oseltamivir as the worst (Figure 4). No heterogeneity was observed (τ^2^ = 0 and *I*^2^ = 0%; 95% CI, 0%-66.5%). There was no measurable global inconsistency based on a random effects design-by-treatment model (χ^2^_2_ = 5.76; *P* = .06) (eTable 2 in the Supplement). We only identified 2 contrasts of inconsistency, indicating disagreement between the indirect and direct evidence for 150 mg oseltamivir vs 75 mg oseltamivir and 150 mg oseltamivir vs placebo (eAppendix 2 in the Supplement). We found that 75 mg oseltamivir was associated with higher occurrence of vomiting compared with placebo (RR, 1.88; 95% CI, 1.47-2.41) ) (eTable 4 in the Supplement). Compared with 75 mg oseltamivir, 300 mg peramivir was associated with a lower frequency of vomiting (RR, 0.32; 95% CI, 0.11-0.93) (eFigure 3 in the Supplement).

### Sensitivity Analysis

A sensitivity analysis was performed by excluding studies with a high risk of bias overall and in the domain of blinding. The overall estimates of TTAS and nausea did not change substantially compared with the base case analysis, indicating that the influence of trials with a high risk of bias was not significant (eTable 3 in the Supplement). The estimates (odds ratio) pooled with the Mantel-Haenszel method were similar to the original comparisons of the antiviral agents with placebo as binary outcomes (eTable 4 in the Supplement).

### Publication Bias

We performed comparison-adjusted funnel plots and Egger test for the primary efficacy outcome, TTAS. Publication bias may present as funnel asymmetry and Egger test (*P* = .049) indicated trials of innovative treatments with favorable trial effects were more likely to get published (eFigure 4 in the Supplement).

### Network Assumptions

We investigated the following factors: age, sex, underlying disease, disease severity, symptom duration until receiving treatment, intervention, treatment duration, concomitant medications, and outcome measures. The characteristics of the studies were mostly similar. The evidence showed there was no substantial heterogeneity or inconsistency in any analysis. However, the possibility of similarity and intransitivity could not be ruled out. Age and disease severity may be associated with modifying associations. Since the similarity of modification cannot be assured, we made the decision to downgrade the certainty of our conclusions owing to transitivity (eTable 6 in the Supplement).^46^

## Discussion

The findings of this network meta-analysis suggest that 10 mg zanamivir twice daily was associated with shorter TTAS compared with placebo. The outcomes for all of the antiviral agents were similar for TTAS, except for 10 mg zanamivir compared with 40 mg or 80 mg baloxavir. Based on moderate-quality evidence, 40 mg or 80 mg baloxavir was associated with fewer complications of influenza compared with placebo.

With respect to safety outcomes, especially gastrointestinal adverse events, 75 mg oseltamivir twice daily, compared with placebo, was associated with more frequent occurrence of nausea based on moderate-quality evidence and of vomiting based on high-quality evidence. Furthermore, compared with oseltamivir, zanamivir and baloxavir were associated with a significantly lower occurrence of nausea, and 300 mg peramivir was associated with a lower frequency of vomiting.

To our knowledge, this is the first study to simultaneously compare the efficacy of baloxavir and 3 neuraminidase inhibitors, the 4 drugs most commonly used for patients with influenza in current clinical practice, using HRs. Compared with a 2019 network meta-analysis by Taieb et al,^71^ we included patients of all ages and a large-scale randomized clinical trial (CAPSTONE-2).^11^ Additionally, we assessed HRs as our treatment outcome in TTAS, while Taieb et al^71^ assessed median values. We also conducted a test of assumption and evaluation of the quality of evidence in this study.

We applied a frequentist framework to perform the analysis. In theory, similar results should be reported under a frequentist framework, as well as a Bayesian framework with noninformative priors.^72^ We also used the common-effect Mantel-Haenszel network meta-analysis, which is helpful to ensure the robustness of evidence when dealing with sparse data.^49^ We included participants of all ages with influenza-like illness; for the primary outcome, TTAS, we only focused on patients with laboratory-confirmed influenza based on a reverse transcription–polymerase chain reaction assay or viral culture with high specificity. The ITT population included patients without confirmed influenza and individuals who may have had poor medication adherence; thus, the analyses may underestimate the efficacy of the antiviral agents.^73^ Therefore, the efficacy analyses of the ITTI population may be closer to the truth than the analyses of the ITT population. Besides, most randomized clinical trials included in this study reported the Kaplan-Meier curves for their ITTI populations, which enabled a more comprehensive comparison of the effectiveness of the drugs.

### Limitations

This network meta-analysis has several limitations. First, because HRs were not fully reported in most studies, our calculations based on reconstruction of the Kaplan-Meier curves may have subtle differences from the actual HRs. Second, there are a lack of studies on some antiviral agents, and the limited number of studies for some treatment comparisons may potentially dominate our network estimates. Third, the definition of complications varied among the studies, which could influence our interpretation of the effectiveness of the drugs for reducing the risk of complications. However, the network meta-analysis mostly identified influenza-related complications, which has a degree of credibility. Due to the lack of standardized definitions of influenza-related complications, meta‐analyses of these outcomes should be interpreted conservatively.^74,75^ Since this network meta-analysis was based on study-level data, the definition of influenza-related complications may be limited to original included trials. Additionally, we investigated all published reports of randomized clinical trials. Although nonrandomized clinical studies were not included, previous nonrandomized clinical studies reported minimal differences in the point estimates compared with our network meta-analysis. Given that nonrandomized clinical studies may be limited by the risk of confounding and selection, reporting, and publication bias, we have reservations about the credibility of their results.^76,77^ Since we did not include regulator reports, the possibility of publication bias cannot be completely ruled out.

## Conclusions

The findings of this network meta-analysis suggest that the examined antiviral agents assessed were associated with shortening TTAS; zanamivir was associated with the lowest TTAS, and baloxavir was associated with the lowest rate of influenza-related complications. Moreover, baloxavir had the lowest risk of total adverse events. Compared with oseltamivir, zanamivir and baloxavir were associated with lower frequencies of nausea and 300 mg peramivir was associated with lower occurrences of vomiting. Network meta-analysis studies such as this may have utility in informing treatment guidelines for viral conditions, like influenza, when few direct comparisons between individual therapies are available.

## Notes

Supplement. **eAppendix 1.** Search Strategy **eTable 1.** Characteristics of Included Studies **eFigure 1.** Risk of Bias of Included Studies **eFigure 2.** Network Graphs of Safety Outcomes **eFigure 3.** League Tables **eTable 2.** Global Inconsistency of All Outcomes **eAppendix 2.** Netsplit Analysis of Inconsistency **eTable 3.** Sensitivity Analysis **eTable 4.** Mantel-Haenszel (MH) Method for Binary Outcomes **eFigure 4.** Comparison-Adjusted Funnel Plot **eTable 5.** Certainty of Direct Evidence Assessment **eTable 6.** Certainty of Network Evidence Assessment
