# Error in Figure Caption

**DOI:** 10.1001/jamanetworkopen.2021.33433

**Published:** 2021-10-06

In the Original Investigation titled “Comparison of Antiviral Agents for Seasonal Influenza Outcomes in Healthy Adults and Children: A Systematic Review and Network Meta-analysis,”^1^ published August 13, 2021, the hazard ratio data in the caption of Figure 3 should have appeared as 0.67 (0.58-0.77) instead of 0.97 (0.73-1.29). This article has been corrected.^1^
