# Supplementary material for: Comparison of Antiviral Agents for Seasonal Influenza Outcomes in Healthy Adults and Children: A Systematic Review and Network Meta-analysis
Source: JAMA Netw Open. 2021 Aug 13;4(8):e2119151. doi: 10.1001/jamanetworkopen.2021.19151 (PMC8363918; doi:10.1001/jamanetworkopen.2021.19151)
Supplement: Supplement. — eAppendix 1. Search Strategy eTable 1. Characteristics of Included Studies eFigure 1. Risk of Bias of Included Studies eFigure 2. Network Graphs of Safety Outcomes eFigure 3. League Tables eTable 2. Global Inconsistency of All Outcomes eAppendix 2. Netsplit Analysis of Inconsistency eTable 3. Sensitivity Analysis eTable 4. Mantel-Haenszel (MH) Method for Binary Outcomes eFigure 4. Comparison-Adjusted Funnel Plot eTable 5. Certainty of Direct Evidence Assessment eTable 6. Certainty of Network Evidence Assessment [file jamanetwopen-e2119151-s001.pdf]

Supplemental Online Content

Liu JW, Lin SH, Wang LC, Chiu HY, Lee JA. Comparison of antiviral agents for seasonal influenza outcomes in healthy adults and children: a systematic review and network meta-analysis. *JAMA Netw Open*. 2021;4(8):e2119151. doi:10.1001/jamanetworkopen.2021.19151

eAppendix 1. Search Strategy
eTable 1. Characteristics of Included Studies
eFigure 1. Risk of Bias of Included Studies
eFigure 2. Network Graphs of Safety Outcomes
eFigure 3. League Tables
eTable 2. Global Inconsistency of All Outcomes
eAppendix 2. Netsplit Analysis of Inconsistency
eTable 3. Sensitivity Analysis
eTable 4. Mantel-Haenszel (MH) Method for Binary Outcomes
eFigure 4. Comparison-Adjusted Funnel Plot
eTable 5. Certainty of Direct Evidence Assessment
eTable 6. Certainty of Network Evidence Assessment

This supplemental material has been provided by the authors to give readers additional information about their work.

© 2021 Liu JW et al. *JAMA Network Open.*

eAppendix 1. Search Strategy

Keywords for randomized-controlled trials

MEDLINE (via PubMed)
1 ("seasonal influenza" OR Influenza, Human [mesh])
2 (oseltamivir OR zanamivir OR tamiflu OR relenza OR peramivir OR rapiacta OR rapivab OR favipiravir OR Avigan OR Pimodivir OR JNJ-63623872 OR VX-787 OR "Baloxavir marboxil" OR "Baloxavir OR S-033188)
3 (randomized controlled trial [pt] OR controlled clinical trial [pt] OR randomized [tiab] OR placebo [tiab] OR drug therapy [sh] OR randomly [tiab] OR trial [tiab] OR groups [tiab])
4 animals [mh] NOT humans [mh]
5 #3 NOT #4
6 #1 AND #2 AND #5

= ("seasonal influenza" OR Influenza, Human [mesh]) AND (oseltamivir OR zanamivir OR tamiflu OR relenza OR peramivir OR rapiacta OR rapivab OR favipiravir OR Avigan OR pimodivir OR JNJ-63623872 OR VX-787 OR "baloxavir marboxil" OR "baloxavir OR S-033188) AND
(randomized controlled trial [pt] OR controlled clinical trial [pt] OR randomized [tiab] OR placebo [tiab] OR drug therapy [sh] OR randomly [tiab] OR trial [tiab] OR groups [tiab]) NOT (animals [mh] NOT humans [mh])

EMBASE (via Embase.com)
1 'influenza'/exp
2 'influenza virus a'/exp OR 'influenza virus b'/de
3 influenza*:ab,ti OR flu:ab,ti
4 #1 OR #2 OR #3
5 'neuraminidase inhibitor':ab,ti OR 'neuraminidase inhibitors':ab,ti
6 'oseltamivir'/de OR 'zanamivir'/de OR 'tamiflu'/de OR 'relenza'/de OR 'peramivir'/de OR 'rapiacta'/de OR 'rapivab'/de OR 'favipiravir'/de OR 'Avigan'/de OR 'Pimodivir'/de OR 'JNJ-63623872'/de OR 'VX-787'/de OR 'Baloxavir marboxil'/de OR 'Baloxavir OR S-033188'/de
7 #5 OR #6
8 #4 AND #7
9 Limit to randomized-controlled trials

('influenza'/exp OR 'influenza virus a'/exp OR 'influenza virus b'/de OR influenza*:ab,ti OR flu:ab,ti) AND ('neuraminidase inhibitor':ab,ti OR 'neuraminidase inhibitors':ab,ti OR 'oseltamivir'/de OR 'zanamivir'/de OR 'tamiflu'/de OR 'relenza'/de OR 'peramivir'/de OR 'rapiacta'/de OR 'rapivab'/de OR 'favipiravir'/de OR 'Avigan'/de OR 'Pimodivir'/de OR 'JNJ-63623872'/de OR 'VX-787'/de OR 'Baloxavir marboxil'/de OR 'Baloxavir OR S-033188'/de) AND ([randomized controlled trial/]lim)

CENTRAL (via Cochrane Library)
(seasonal influenza OR [mh "influenza, human"]) AND (oseltamivir OR zanamivir OR tamiflu OR relenza OR peramivir OR rapiacta OR rapivab OR favipiravir OR Avigan OR Pimodivir OR JNJ-63623872 OR VX-787 OR "Baloxavir marboxil" OR Baloxavir OR S-033188)

© 2021 Liu JW et al. *JAMA Network Open.*

Keywords for systematic review/meta-analysis

MEDLINE (via PubMed)
1 ("seasonal influenza" OR Influenza, Human [mesh])
2 (oseltamivir OR zanamivir OR tamiflu OR relenza OR peramivir OR rapiacta OR rapivab OR favipiravir OR Avigan OR Pimodivir OR JNJ-63623872 OR VX-787 OR "Baloxavir marboxil" OR Baloxavir OR S-033188)
3 systematic [sb]
4 #1 AND #2 AND #3

EMBASE (via Embase.com)
1 'influenza'/exp
2 'influenza virus a'/exp OR 'influenza virus b'/de
3 influenza*:ab,ti OR flu:ab,ti
4 #1 OR #2 OR #3
5 'neuraminidase inhibitor':ab,ti OR 'neuraminidase inhibitors':ab,ti
6 'oseltamivir'/de OR 'zanamivir'/de OR 'tamiflu'/de OR 'relenza'/de OR 'peramivir'/de OR 'rapiacta'/de OR 'rapivab'/de OR 'favipiravir'/de OR 'Avigan'/de OR 'Pimodivir'/de OR 'JNJ-63623872'/de OR 'VX-787'/de OR 'Baloxavir marboxil'/de OR 'Baloxavir OR S-033188'/de
7 #5 OR #6
8 #4 AND #7
9 Limit to systematic review

CENTRAL (via Cochrane Library)
(seasonal influenza OR [mh "influenza, human"]) AND (oseltamivir OR zanamivir OR tamiflu OR relenza OR peramivir OR rapiacta OR rapivab OR favipiravir OR Avigan OR Pimodivir OR JNJ-63623872 OR VX-787 OR "Baloxavir marboxil" OR Baloxavir OR S-033188)
limit to Cochrane review

© 2021 Liu JW et al. *JAMA Network Open.*

eTable 1. Characteristics of Included Studies

Studies included in **qualitative and quantitative** synthesis (network meta-analysis) (n=26)									
Trial, published year	Country	Population	Group (age)	Intervention	Drugs		Control		Outcomes
					ITT (No. randomized)	ITTI (No. confirmed)	ITT	ITTI	
Deng,2004	China (published in Chinese)	ILI (≤ 48h)	16-48	OSE 75 mg BID<br>PLA	599	NA	577	NA	TTAS **(only ITT data reported)**<br>Complications: sinusitis, tracheobronchitis, tonsillitis, bronchial asthma, otitis media, etc.<br>AEs: GI, dizziness, etc.
Fan,2019	China (published in Chinese)	ILI (≤ 48h)<br>RAT(+)	15-70	PER 600 mg single dose<br>OSE 75 mg BID<br>PLA	PER: 50<br>OSE: 57	PER: 47<br>OSE: 53	26	26	TTAS **(without ITTI HR or KM-curve)**<br>Complications: Otitis media, sinusitis, pneumonia, etc.<br>AEs: no detail definition mentioned.
Fry,2014	Bangladesh	ILI (≤ 120h)<br>RAT(+)	All<br>(≥1)	OSE 75 mg BID<br>PLA	598	587	592	576	TTAS **(only ITT data reported)**<br>Complication: Not reported.<br>AEs: GI、severe AE (requiring hospital admission)
Hayden,1997	North America, Europe	ILI (≤ 48h)	Adults<br>(≥18)	ZAN 10 mg BID<br>PLA	132	85	144	89	TTAS (ITTI)<br>Complication (antibiotics-prescribed) otitis media, sinusitis, etc.<br>AEs: GI, upper respiratory tract, etc.
Hayden,2018	Japan	ILI (≤ 48h)	12-64	BAL 40/80 single dose<br>OSE 75 mg BID<br>PLA	BAL: 612<br>OSE: 514	BAL: 456<br>OSE: 377	PLA: 310	PLA: 231	TTAS (ITTI)<br>Complications (antibiotic use)<br>AEs: GI, nasopharyngitis, liver function, headache, dizziness, leukopenia, etc.
Hayden,2000	US, Canada, UK, Finland	ILI (≤ 36h)	≥5	ZAN 10 mg BID<br>PLA	163	76	158	81	TTAS **(without ITTI HR or KM-curve)**<br>Complications: Not reported.<br>AEs: no detail definition mentioned (but including pneumonia).
Hedrick, 2000	US, Canada, Europe, Israel	ILI (≤ 36h)	**Children (5-12)**	ZAN 10 mg BID<br>PLA	224	164	247	182	TTAS (ITTI)<br>Complications: no detail definition mentioned.<br>AEs: GI, respiratory disorder, laboratory abnormalities.

© 2021 Liu JW et al. *JAMA Network Open.*

Heinonen,2010	Finland	ILI (≤24h)	**Children (1-3)**	OSE 75 mg BID<br>PLA	203	37	205	61	TTAS (ITTI)<br>Complications: only acute otitis media, pneumonia reported.<br>AEs: GI, bronchiolitis, exanthema, irritability, fatigue, headache, or decreased appetite, etc.
Ison,2020	Japan, South Korea, Taiwan, US, Europe, NZ, South Africa (17 countries)	ILI (≤ 48h)	≥12	BAL single dose<br>OSE 75 mg BID<br>PLA	BAL: 730<br>OSE: 725	BAL: 385<br>OSE:388	729	385	TTAS<br>Complications: sinusitis, otitis media, bronchitis, pneumonia.<br>AEs: bronchitis, sinusitis, diarrhea, nausea, etc.
Jong,2014	US, Canada, South America, Europe, India, NZ, South Africa (21 countries)	**Hospitalized**<br>RAT(+)	≥6	PER 600 mg QD (5 days)<br>PLA	NA	78	NA	43	TTAS **(without ITTI HR or KM-curve)**<br>Complications: otitis, sinusitis, bronchitis, **pneumonia**.<br>AEs: GI, serious AEs (COPD, **pneumonia**)
Kashiwagi,2000	Japan (published in Japanese)	ILI (≤ 36h)	≥16	OSE 75 mg BID<br>PLA	154	121	162	130	TTAS (ITTI)<br>Complications: NA<br>AEs: GI, infection (**pneumonia**), musculoskeletal disorders, respiratory disorders, skin and subcutaneous disorders, metabolism disorders, etc.
Kohno,2010	Japan	ILI (≤ 48h)	Adults (20-64)	PER 300 mg single dose<br>PER 600 mg single dose<br>PLA	PER 300: 100<br>PER 600: 100	PER 300: 99<br>PER 600: 97	100	100	TTAS (ITTI)<br>Complications: otitis media, bronchitis, sinusitis, pneumonia.<br>AEs: GI, nasopharyngitis, liver function disorders, hematology disorders, metabolism disorders, etc.
Kohno,2011	Japan	ILI (≤ 48h)<br>RAT(+)	Adults (≥20)	PER 300 mg QD<br>PER 600 mg QD (1-5 days as needed)	PER 300: 21	NA	PER 600: 21	NA	TTAS **(only per-protocol data)**<br>Complications: **pneumonia**, bronchitis, otitis media, sinusitis.<br>AEs: GI, **pneumonia**, oral herpes infection, hematology disorders, etc.

© 2021 Liu JW et al. *JAMA Network Open.*

Li,2004	China	LI (≤ 36h)	Adults (18-65)	OSE 75 mg BID<br>PLA	216	134	235	139	TTAS (ITTI)<br>Complications: bronchitis, pneumonia, sinusitis, tonsillitis, otitis media, etc.<br>AEs: GI, neurological symptoms, rashes, etc.
Lin,2006	China	ILI (≤ 48h)	Adults (OSE: 48.1±0.8 S: 52.3±16.0)	OSE 75 mg BID<br>Symptomatic treatment	58	27	60	29	TTAS (ITTI)<br>Complications: tracheitis, bronchitis, pneumonia, nasosinusitis, pharyngitis.<br>AEs: GI.
Mäkelä,2000	Europe	ILI (≤ 48h)	≥12	ZAN 10 mg BID<br>PLA	174	136	182	141	TTAS (ITTI)<br>Complications: generally pulmonary, infections.<br>AEs: GI, bronchitis, sinusitis, **pneumonia**, etc.
MIST,1998	Australia, New Zealand, South Africa	ILI (≤ 36h)	≥12	ZAN 10 mg BID<br>PLA	227	161	228	160	TTAS **(without ITTI HR or KM-curve)**<br>Complications: bronchitis, pneumonia, chest infections, etc.<br>AEs: GI, bronchitis, cough, sinusitis, LRTI, etc.
Monto,1999	Not reported	ILI (≤ 48h)	All	ZAN 10 mg BID<br>PLA	1133	807	1102	765	TTAS **(without ITTI HR or KM-curve)**<br>Complications: (antibiotic use)<br>sinusitis, otitis media, bronchitis, etc.<br>AEs: NA
Nakamura,2017	Japan	ILI (≤ 48h)<br>RAT(+)	Adults (≥ 20)	PER 600 mg single dose<br>OSE 75 mg BID	PER: 46	as ITT	OSE: 46	as ITT	TTAS **(only alleviation of fever reported)**<br>Complications: pneumonia, bronchitis, bronchial asthma attack, etc.<br>AEs: GI, **pneumonia**, bronchial asthma attack, COPD exacerbation, liver dysfunction, decrease WBC counts, etc.
Nicholson,2000	Europe, Canada	ILI (≤ 36h)	Adults (18-65)	OSE 75 mg BID<br>OSE 150 mg BID<br>PLA	OSE 75: 241<br>OSE 150: 243	OSE 75: 158<br>OSE 150: 156	PLA: 235	PLA: 161	TTAS (ITTI)<br>Complications (antibiotic treatment): otitis media, bronchitis, sinusitis, pneumonia.<br>AEs: GI.
Puhakka,2003	Finland	ILI (≤ 48h)	All	ZAN 10 mg BID<br>PLA	293	222	295	213	TTAS **(without ITTI HR or KM-curve)**<br>Complications: no detail definition mentioned.<br>AEs: GI, sinusitis, tonsillitis, pneumonia,

© 2021 Liu JW et al. *JAMA Network Open.*

									hemorrhage, musculoskeletal pain, fever, cough, etc.
SEAICRN,2013	Indonesia, Singapore, Thailand, Vietnam	**Hospitalized** RAT (+)	All (≥1)	OSE 75 mg BID<br>OSE 150 mg BID	OSE 150: 165	OSE 150: 159	OSE 75: 161	OSE 75: 154	TTAS, Complication: Not reported.<br>AEs: GI, respiratory failure, multi-organ failure, bronchitis, pneumothorax, neutropenia, thrombocytosis, pyrexia, sepsis, rash, etc.
Treanor,2000	US	ILI (≤ 36h)	Adults (18-65)	OSE 75 mg BID<br>OSE 150 mg BID<br>PLA	OSE 75: 211<br>OSE 150: 209	OSE 75: 124<br>OSE 150: 121	PLA: 209	PLA: 129	TTAS (ITTI)<br>Complications: otitis, bronchitis, sinusitis, and pneumonia<br>AEs: GI
Watanabe,2019	Japan	Influenza-confirmed RAT(+) & PCR	Adults (20-65)	BAL 10 mg<br>BAL 20 mg<br>BAL 40 mg single<br>PLA	BAL 10: 100<br><br>BAL 20: 100<br><br>BAL 40: 100	as ITT	100	as ITT	TTAS (ITTI)<br>Complications: sinusitis, otitis media, bronchitis, pneumonia.<br>AEs: GI, nasopharyngitis, headache, liver function disorder, WBC decreased, etc.
Whitley,2001	US, Canada	ILI (≤ 48h)	**Children (1-2)**	OSE 75 mg BID<br>PLA	344	217	351	235	TTAS (ITTI)<br>Complications: otitis media, sinusitis, bronchitis or pneumonia.<br>AEs: GI, **pneumonia**, dehydration, etc.
Whitley,2015	US	ILI (≤ 48h)<br>RAT(+)	Adults (≥18)	PER 300 mg single dose<br>PLA	172	163	141	134	TTAS (ITTI)<br>Complications: sinusitis, otitis, bronchitis, pneumonia.<br>AEs: GI, renal/urinary disorders, dizziness, infections, respiratory disorders, myalgia, metabolism disorders, insomnia
Studies included in **only qualitative** synthesis (without NMA)									
Boivin,2000	Canada	ILI	≥12	ZAN 10 mg BID<br>PLA	NA	17	NA	10	TTAS **(without ITTI HR or KM-curve)**<br>Complications, AEs: Not reported.
Ison,2013	Australia, Canada, HK, NZ, South Africa, US	**Hospitalized** RAT(+)	Adults (≥18)	OSE 75 mg BID<br><br>PER 200/400 (not our interest)	46	41	PER 200/400	PER 200/400	TTAS, Complications: Not reported.<br>AEs: GI, hypokalemia, myocarditis, **pneumonia**, etc.

© 2021 Liu JW et al. *JAMA Network Open.*

Katsumi,2012	Japan	ILI (≤ 48h)	**Children (≤15)**	LAN 20/40 mg single dose (not our interest)<br>ZAN 10 mg BID	LAN: 55	NA	ZAN: 57	NA	TTAS **(without ITTI HR or KM-curve)**<br>Complications: asthmatic symptoms, pneumonia.<br>AEs: GI, abnormal behaviors.
Ison,2014	US, Canada, Mexico	**Hospitalized**	≥6	PER 600 mg QD (5 days)<br>PER 300 mg BID (5 days)	PER 600: 117	70	PER 300: 117	57	TTAS **(without ITTI HR or KM-curve)**<br>Complications: otitis, sinusitis, bronchitis, pneumonia.<br>AEs: GI, metabolism disorders, edema, headache, agitation, insomnia, etc.
Matsumoto,1999	Japan	ILI (≤ 36h)	16-65	ZAN 10 mg BID<br>PLA	37	22	39	29	TTAS **(without ITTI HR or KM-curve)**<br>Complications: NA<br>AEs: any laboratory abnormalities.
Sugaya,2010	Japan	ILI (≤ 36h)	**Children (≤9)**	LAN 20/40 mg single dose<br>OSE 75 mg BID	LAN 40: 62<br><br>LAN 20: 62	NA	OSE 75: 62	NA	TTAS **(with only ITT data)**<br>Complications: Not reported.<br>AEs: GI, gastroenteritis.
Watanabe,2010	Europe, North America	ILI (≤ 36h)<br>RAT (+)	Adults (≥20)	LAN 20/40 mg single dose (not our interest)<br><br>OSE 75 mg BID	LAN 40: 338<br>LAN 20: 327	NA	OSE 75: 338	NA	TTAS: **NA** (**with** only ITT data)<br>Complications: NA<br>AEs: GI, dizziness

PLA = placebo, BAL 40/80 = baloxavir 40 or 80 mg, OSE 75 = oseltamivir 75 mg, OSE 150 = oseltamivir 150 mg, PER 300 = peramivir 300 mg, PER 600 = peramivir 600 mg, ZAN 10 = zanamivir 10 mg, LAN 20/40 = laninamivir 20 or 40 mg
ILI = influenza-like illness, RAT = rapid antigen test
ITT = intention-to-treat, ITTI = intention-to-treat-infected

© 2021 Liu JW et al. *JAMA Network Open.*

eFigure 1. Risk of Bias of Included Studies

Risk of bias graph

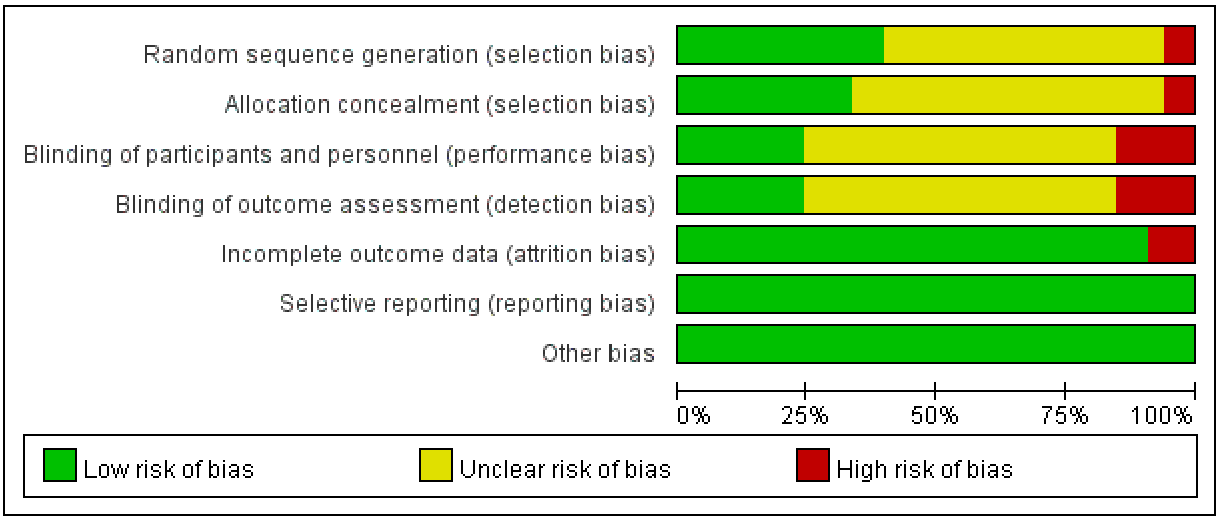

Risk of bias summary

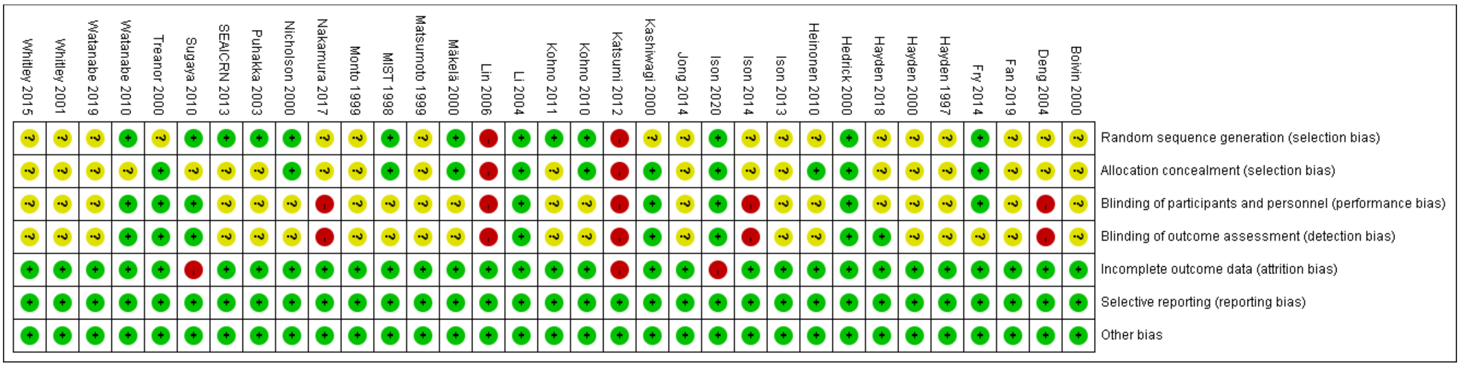

Overall, 2 trials were judged to have a low risk of bias across all domains.
2 trials were judged to have a high risk of bias for sequence generation and allocation concealment due to inadequate randomization and assignments.
5 trials were open-labeled and therefore judged to have a high risk of performance bias.
Most studies were judged to have a low risk of bias for incomplete outcome data and selective reporting; however, 3 trials were judged to have a high risk of bias for incomplete outcome data, as drop-outs and loss to follow-up could have a clinically relevant impact on the intervention effect estimates.

© 2021 Liu JW et al. *JAMA Network Open.*

eFigure 2. Network Graphs of Safety Outcomes

A. Total adverse events

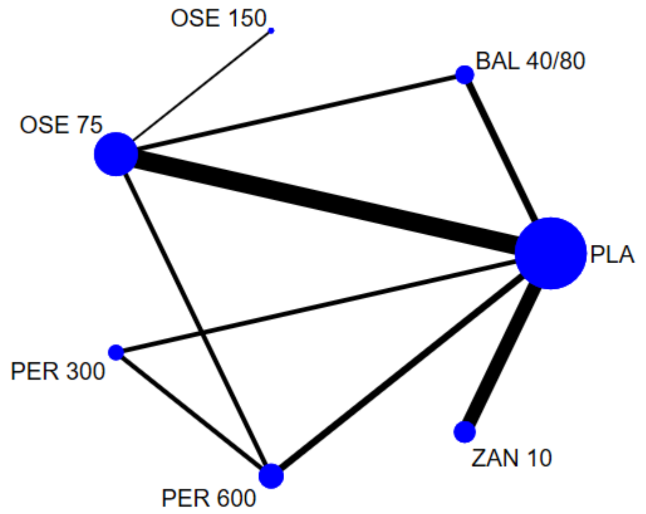

B. Nausea

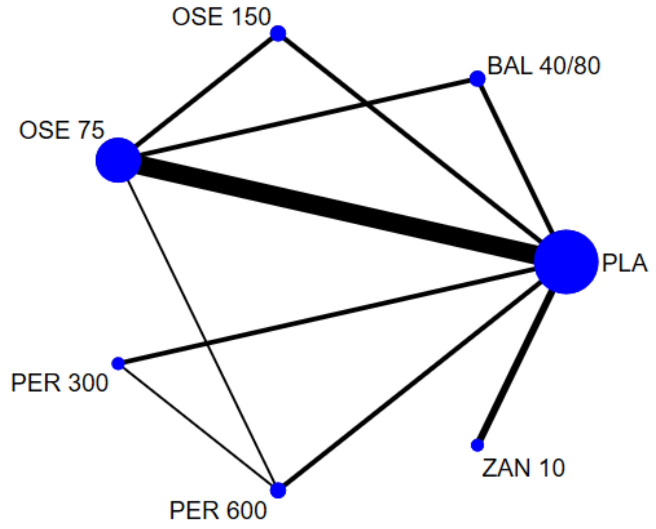

C. Vomiting

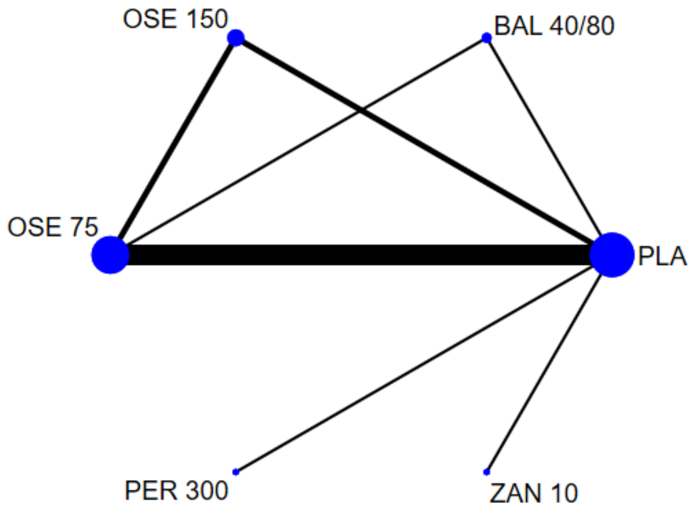

PLA=placebo, BAL 40/80=baloxavir 40 or 80 mg, OSE 75=oseltamivir 75 mg, OSE 150=oseltamivir 150 mg, PER 300=peramivir 300 mg, PER 600=peramivir 600 mg, ZAN 10=zanamivir 10 mg

© 2021 Liu JW et al. *JAMA Network Open.*

eFigure 3. League Tables

Effect sizes presented on the upper triangle are direct comparisons (head-to-head studies) between the row and columns; the effect sizes on the lower triangle are network meta-analyses between the column and the row. Comparisons are based on hazard ratio (95% confidence intervals) in TTAS and risk ratio (95% confidence intervals) in other outcomes.

A. TTAS

**Zanamivir 10 mg**	.	.	.	.	.	**0.67 (0.58-0.77)**
0.97 (0.73-1.29)	**Peramivir 600 mg**	.	.	0.99 (0.75-1.30)	.	**0.64 (0.48-0.84)**
0.90 (0.77-1.05)	0.93 (0.71-1.20)	**Oseltamivir 75 mg**	0.98 (0.83-1.16)	.	0.96 (0.87-1.05)	**0.74 (0.69-0.79)**
0.90 (0.73-1.09)	0.92 (0.69-1.23)	1.00 (0.86-1.15)	**Oseltamivir 150 mg**	.	.	**0.74 (0.63-0.86)**
0.89 (0.70-1.13)	0.92 (0.71-1.18)	0.99 (0.81-1.21)	0.99 (0.78-1.26)	**Peramivir 300 mg**	.	**0.75 (0.62-0.91)**
**0.84 (0.71-0.99)**	0.87 (0.67-1.13)	0.94 (0.86-1.02)	0.94 (0.80-1.10)	0.95 (0.77-1.17)	**Baloxavir 40 or 80 mg**	**0.81 (0.74-0.89)**
**0.67 (0.58-0.77)**	**0.69 (0.54-0.88)**	**0.74 (0.70-0.79)**	**0.75 (0.65-0.86)**	**0.75 (0.62-0.91)**	**0.79 (0.73-0.86)**	**Placebo**

B. Complications

**Baloxavir 40 or 80 mg**	0.91 (0.53-1.57)	.	.	.	.	**0.44 (0.27-0.72)**
0.83 (0.52-1.32)	**Oseltamivir 75 mg**	1.07 (0.63-1.80)	1.62 (0.53-4.95)	.	.	**0.61 (0.49-0.75)**
0.78 (0.41-1.46)	0.94 (0.59-1.48)	**Oseltamivir 150 mg**	.	.	.	0.69 (0.42-1.12)
0.77 (0.37-1.59)	0.93 (0.52-1.68)	0.99 (0.48-2.04)	**Peramivir 600 mg**	0.63 (0.15-2.61)	.	0.82 (0.41-1.64)
0.75 (0.38-1.49)	0.91 (0.52-1.57)	0.97 (0.49-1.92)	0.97 (0.47-2.00)	**Peramivir 300 mg**	.	0.65 (0.38-1.11)
**0.62 (0.39-0.99)**	**0.75 (0.59-0.95)**	0.80 (0.50-1.27)	0.80 (0.45-1.43)	0.82 (0.49-1.39)	**Zanamivir 10 mg**	**0.82 (0.72-0.92)**
**0.51 (0.32-0.80)**	**0.61 (0.49-0.75)**	0.65 (0.41-1.02)	0.65 (0.37-1.16)	0.67 (0.40-1.12)	**0.82 (0.72-0.92)**	**Placebo**

© 2021 Liu JW et al. *JAMA Network Open.*

C. Total adverse events

**Baloxavir 40 or 80 mg**	.	.	.	0.87 (0.76-1.00)	.	**0.85 (0.74-0.97)**
0.96 (0.80-1.14)	**Zanamivir 10 mg**	.	.	.	.	0.88 (0.78-1.00)
0.89 (0.77-1.04)	0.93 (0.80-1.08)	**Peramivir 300 mg**	.	.	0.96 (0.88-1.06)	0.96 (0.88-1.05)
0.86 (0.53-1.42)	0.90 (0.54-1.50)	0.97 (0.59-1.60)	**Oseltamivir 150 mg**	1.01 (0.62-1.64)	.	.
**0.87 (0.77-0.99)**	0.91 (0.78-1.07)	0.98 (0.86-1.12)	1.01 (0.62-1.64)	**Oseltamivir 75 mg**	1.61 (0.83-3.12)	0.95 (0.86-1.05)
0.87 (0.75-1.01)	0.91 (0.78-1.05)	0.98 (0.89-1.07)	1.01 (0.61-1.65)	0.99 (0.88-1.13)	**Peramivir 600 mg**	0.97 (0.90-1.06)
**0.84 (0.74-0.96)**	0.88 (0.78-1.00)	0.95 (0.87-1.03)	0.97 (0.60-1.59)	0.96 (0.87-1.06)	0.97 (0.90-1.05)	**Placebo**

D. Nausea

**Zanamivir 10 mg**	.	0.55 (0.26-1.17)	.	.	.	.
0.64 (0.27-1.56)	**Baloxavir 40 or 80 mg**	0.74 (0.44-1.22)	.	.	**0.53 (0.33-0.83)**	.
0.55 (0.26-1.17)	0.85 (0.54-1.34)	**Placebo**	0.77 (0.35-1.65)	0.92 (0.42-2.03)	**0.55 (0.42-0.73)**	**0.36 (0.23-0.56)**
0.54 (0.19-1.52)	0.83 (0.36-1.95)	0.98 (0.48-2.00)	**Peramivir 300 mg**	0.50 (0.13-1.94)	.	.
0.48 (0.17-1.34)	0.74 (0.32-1.70)	0.87 (0.43-1.75)	0.89 (0.37-2.13)	**Peramivir 600 mg**	0.33 (0.01-7.97)	.
**0.30 (0.13-0.67)**	**0.47 (0.30-0.72)**	**0.55 (0.42-0.72)**	0.56 (0.26-1.21)	0.63 (0.30-1.34)	**Oseltamivir 75 mg**	0.94 (0.68-1.28)
**0.26 (0.11-0.60)**	**0.40 (0.24-0.67)**	**0.47 (0.33-0.67)**	0.48 (0.22-1.07)	0.54 (0.25-1.19)	0.86 (0.63-1.16)	**Oseltamivir 150 mg**

E. Vomiting

**Peramivir 300 mg**	0.61 (0.22-1.71)	.	.	.	.
0.61 (0.22- 1.71)	**Placebo**	0.91 (0.32-2.55)	0.79 (0.15-4.05)	**0.53 (0.41-0.68)**	**0.27 (0.15-0.48)**
0.55 (0.13- 2.37)	0.91 (0.32- 2.55)	**Zanamivir 10 mg**	.	.	.
0.46 (0.10- 2.15)	0.77 (0.25- 2.37)	0.84 (0.18- 3.90)	**Baloxavir 40 or 80 mg**	0.70 (0.22-2.28)	.
**0.32 (0.11- 0.93)**	**0.53 (0.41- 0.68)**	0.59 (0.20- 1.69)	0.69 (0.23- 2.11)	**Oseltamivir 75 mg**	0.96 (0.67-1.37)
**0.27 (0.09- 0.82)**	**0.45 (0.30- 0.66)**	0.49 (0.16- 1.49)	0.58 (0.18- 1.87)	0.84 (0.60- 1.19)	**Oseltamivir 150 mg**

© 2021 Liu JW et al. *JAMA Network Open.*

eTable 2. Global Inconsistency of All Outcomes

Outcome	Q score	P-value	tau.within	tau2.within
TTAS	2.37 (df=4)	0.6689	0.0322	0.0010
Complications	5.05 (df=9)	0.8302	0.0895	0.0080
Total adverse events	6.42 (df=8)	0.6007	0	0
Nausea	7.89 (df=5)	0.1626	0	0
Vomiting	5.76 (df=2)	0.0562	0	0

© 2021 Liu JW et al. *JAMA Network Open.*

eAppendix 2. Netsplit Analysis of Inconsistency

TTAS

```
Random effects model
comparison - Treatment comparison
k           - Number of studies providing direct evidence
prop        - Direct evidence proportion
nma         - Estimated treatment effect (HR) in network meta-analysis
direct      - Estimated treatment effect (HR) derived from direct evidence
indir.      - Estimated treatment effect (HR) derived from indirect evidence
RoR         - Ratio of Ratios (direct versus indirect)
z           - z-value of test for disagreement (direct versus indirect)
p-value      - p-value of test for disagreement (direct versus indirect)
```

Comparison	k	prop	nma	95%-CI	direct	95%-CI	indir.	95%-CI	RoR	95%-CI	z	p-value
BAL 40/80:OSE 150	0	0	1.06	(0.91- 1.24)	.	.	1.06	(0.91- 1.24)	.	.	.	.
BAL 40/80:OSE 75	2	0.85	1.07	(0.98- 1.16)	1.05	(0.96- 1.14)	1.20	(0.97- 1.48)	0.87	(0.69- 1.10)	-1.15	0.2504
BAL 40/80:PER 300	0	0	1.06	(0.86- 1.30)	.	.	1.06	(0.86- 1.30)	.	.	.	.
BAL 40/80:PER 600	0	0	1.15	(0.88- 1.50)	.	.	1.15	(0.88- 1.50)	.	.	.	.
BAL 40/80:PLA	3	0.84	0.79	(0.73- 0.86)	0.81	(0.74- 0.89)	0.71	(0.58- 0.88)	1.13	(0.90- 1.42)	1.06	0.2906
BAL 40/80:ZAN 10	0	0	1.19	(1.01- 1.40)	.	.	1.19	(1.01- 1.40)	.	.	.	.
OSE 150:OSE 75	2	0.74	1.00	(0.87- 1.16)	1.02	(0.86- 1.20)	0.96	(0.73- 1.27)	1.06	(0.77- 1.47)	0.37	0.7141
OSE 150:PER 300	0	0	0.99	(0.78- 1.26)	.	.	0.99	(0.78- 1.26)	.	.	.	.
OSE 150:PER 600	0	0	1.08	(0.81- 1.44)	.	.	1.08	(0.81- 1.44)	.	.	.	.
OSE 150:PLA	2	0.78	0.75	(0.65- 0.86)	0.74	(0.63- 0.86)	0.78	(0.58- 1.06)	0.95	(0.67- 1.33)	-0.32	0.7479
OSE 150:ZAN 10	0	0	1.12	(0.91- 1.36)	.	.	1.12	(0.91- 1.36)	.	.	.	.
OSE 75:PER 300	0	0	0.99	(0.81- 1.21)	.	.	0.99	(0.81- 1.21)	.	.	.	.
OSE 75:PER 600	0	0	1.08	(0.83- 1.40)	.	.	1.08	(0.83- 1.40)	.	.	.	.
OSE 75:PLA	9	0.98	0.74	(0.70- 0.79)	0.74	(0.69- 0.79)	0.99	(0.62- 1.56)	0.75	(0.47- 1.19)	-1.23	0.2195
OSE 75:ZAN 10	0	0	1.11	(0.95- 1.30)	.	.	1.11	(0.95- 1.30)	.	.	.	.
PER 300:PER 600	1	0.84	1.09	(0.85- 1.40)	1.01	(0.77- 1.33)	1.61	(0.87- 2.99)	0.63	(0.32- 1.24)	-1.35	0.1778
PER 300:PLA	2	1.00	0.75	(0.62- 0.91)	0.75	(0.62- 0.91)	.	.	.	.	.	.
PER 300:ZAN 10	0	0	1.12	(0.88- 1.43)	.	.	1.12	(0.88- 1.43)	.	.	.	.
PER 600:PLA	1	0.84	0.69	(0.54- 0.88)	0.64	(0.48- 0.84)	1.02	(0.55- 1.89)	0.63	(0.32- 1.24)	-1.35	0.1778
PER 600:ZAN 10	0	0	1.03	(0.77- 1.37)	.	.	1.03	(0.77- 1.37)	.	.	.	.
PLA:ZAN 10	3	1.00	1.50	(1.30- 1.73)	1.50	(1.30- 1.73)	.	.	.	.	.	.

© 2021 Liu JW et al. *JAMA Network Open.*

Complications

Random effects model
comparison - Treatment comparison
k - Number of studies providing direct evidence
prop - Direct evidence proportion
nma - Estimated treatment effect (HR) in network meta-analysis
direct - Estimated treatment effect (HR) derived from direct evidence
indir. - Estimated treatment effect (HR) derived from indirect evidence
RoR - Ratio of Ratios (direct versus indirect)
z - z-value of test for disagreement (direct versus indirect)
p-value - p-value of test for disagreement (direct versus indirect)

comparison	k	prop	nma	95%-CI	direct	95%-CI	indir.	95%-CI	RoR	95%-CI	z	p-value
BAL 40/80:OSE 150	0	0	0.78	(0.41- 1.46)	.	.	0.78	(0.41- 1.46)	.	.	.	.
BAL 40/80:OSE 75	2	0.74	0.83	(0.52- 1.32)	0.91	(0.53- 1.57)	0.63	(0.25- 1.58)	1.45	(0.50- 4.21)	0.68	0.4957
BAL 40/80:PER 300	0	0	0.75	(0.38- 1.49)	.	.	0.75	(0.38- 1.49)	.	.	.	.
BAL 40/80:PER 600	0	0	0.77	(0.37- 1.59)	.	.	0.77	(0.37- 1.59)	.	.	.	.
BAL 40/80:PLA	3	0.86	0.51	(0.32- 0.80)	0.44	(0.27- 0.72)	1.17	(0.35- 3.94)	0.38	(0.10- 1.40)	-1.46	0.1441
BAL 40/80:ZAN 10	0	0	0.62	(0.39- 0.99)	.	.	0.62	(0.39- 0.99)	.	.	.	.
OSE 150:OSE 75	2	0.78	1.07	(0.67- 1.69)	0.94	(0.56- 1.58)	1.71	(0.64- 4.55)	0.55	(0.18- 1.67)	-1.06	0.2904
OSE 150:PER 300	0	0	0.97	(0.49- 1.92)	.	.	0.97	(0.49- 1.92)	.	.	.	.
OSE 150:PER 600	0	0	0.99	(0.48- 2.04)	.	.	0.99	(0.48- 2.04)	.	.	.	.
OSE 150:PLA	2	0.86	0.65	(0.41- 1.02)	0.69	(0.42- 1.12)	0.45	(0.13- 1.53)	1.53	(0.41- 5.70)	0.64	0.5250
OSE 150:ZAN 10	0	0	0.80	(0.50- 1.27)	.	.	0.80	(0.50- 1.27)	.	.	.	.
OSE 75:PER 300	0	0	0.91	(0.52- 1.57)	.	.	0.91	(0.52- 1.57)	.	.	.	.
OSE 75:PER 600	2	0.28	0.93	(0.52- 1.68)	1.62	(0.53- 4.95)	0.75	(0.38- 1.51)	2.14	(0.57- 7.99)	1.13	0.2570
OSE 75:PLA	9	0.97	0.61	(0.49- 0.75)	0.61	(0.49- 0.75)	0.74	(0.21- 2.62)	0.82	(0.23- 2.93)	-0.31	0.7544
OSE 75:ZAN 10	0	0	0.75	(0.59- 0.95)	.	.	0.75	(0.59- 0.95)	.	.	.	.
PER 300:PER 600	2	0.25	1.03	(0.50- 2.11)	1.60	(0.38- 6.66)	0.88	(0.38- 2.03)	1.81	(0.35- 9.45)	0.70	0.4826
PER 300:PLA	2	0.92	0.67	(0.40- 1.12)	0.65	(0.38- 1.11)	0.95	(0.15- 6.00)	0.68	(0.10- 4.63)	-0.39	0.6966
PER 300:ZAN 10	0	0	0.82	(0.49- 1.39)	.	.	0.82	(0.49- 1.39)	.	.	.	.
PER 600:PLA	3	0.68	0.65	(0.37- 1.16)	0.82	(0.41- 1.64)	0.40	(0.15- 1.10)	2.05	(0.61- 6.94)	1.15	0.2489
PER 600:ZAN 10	0	0	0.80	(0.45- 1.43)	.	.	0.80	(0.45- 1.43)	.	.	.	.
ZAN 10:PLA	6	1.00	0.82	(0.72- 0.92)	0.82	(0.72- 0.92)	.	.	.	.	.	.

© 2021 Liu JW et al. *JAMA Network Open.*

Total adverse events

Random effects model
comparison - Treatment comparison
k - Number of studies providing direct evidence
prop - Direct evidence proportion
nma - Estimated treatment effect (HR) in network meta-analysis
direct - Estimated treatment effect (HR) derived from direct evidence
indir. - Estimated treatment effect (HR) derived from indirect evidence
RoR - Ratio of Ratios (direct versus indirect)
z - z-value of test for disagreement (direct versus indirect)
p-value - p-value of test for disagreement (direct versus indirect)

comparison	k	prop	nma	95%-CI	direct	95%-CI	indir.	95%-CI	RoR	95%-CI	z	p-value
BAL 40/80:OSE 150	0	0	0.86	(0.53- 1.42)	.	.	0.86	(0.53- 1.42)	.	.	.	.
BAL 40/80:OSE 75	2	0.87	0.87	(0.77- 0.99)	0.87	(0.76- 1.00)	0.90	(0.64- 1.27)	0.97	(0.67- 1.40)	-0.18	0.8597
BAL 40/80:PER 300	0	0	0.89	(0.77- 1.04)	.	.	0.89	(0.77- 1.04)	.	.	.	.
BAL 40/80:PER 600	0	0	0.87	(0.75- 1.01)	.	.	0.87	(0.75- 1.01)	.	.	.	.
BAL 40/80:PLA	3	0.88	0.84	(0.74- 0.96)	0.85	(0.74- 0.97)	0.81	(0.57- 1.17)	1.04	(0.71- 1.53)	0.21	0.8335
BAL 40/80:ZAN 10	0	0	0.96	(0.80- 1.14)	.	.	0.96	(0.80- 1.14)	.	.	.	.
OSE 150:OSE 75	1	1.00	1.01	(0.62- 1.64)	1.01	(0.62- 1.64)	.	.	.	.	.	.
OSE 150:PER 300	0	0	1.03	(0.63- 1.70)	.	.	1.03	(0.63- 1.70)	.	.	.	.
OSE 150:PER 600	0	0	1.01	(0.61- 1.65)	.	.	1.01	(0.61- 1.65)	.	.	.	.
OSE 150:PLA	0	0	0.97	(0.60- 1.59)	.	.	0.97	(0.60- 1.59)	.	.	.	.
OSE 150:ZAN 10	0	0	1.11	(0.67- 1.84)	.	.	1.11	(0.67- 1.84)	.	.	.	.
OSE 75:PER 300	0	0	1.02	(0.89- 1.16)	.	.	1.02	(0.89- 1.16)	.	.	.	.
OSE 75:PER 600	2	0.04	0.99	(0.88- 1.13)	1.61	(0.83- 3.12)	0.98	(0.86- 1.11)	1.65	(0.84- 3.24)	1.45	0.1460
OSE 75:PLA	8	0.97	0.96	(0.87- 1.06)	0.95	(0.86- 1.05)	1.52	(0.83- 2.78)	0.62	(0.34- 1.15)	-1.51	0.1316
OSE 75:ZAN 10	0	0	1.09	(0.93- 1.28)	.	.	1.09	(0.93- 1.28)	.	.	.	.
PER 300:PER 600	2	0.93	0.98	(0.89- 1.07)	0.96	(0.88- 1.06)	1.12	(0.80- 1.57)	0.86	(0.61- 1.22)	-0.86	0.3884
PER 300:PLA	2	0.95	0.95	(0.87- 1.03)	0.96	(0.88- 1.05)	0.72	(0.48- 1.07)	1.34	(0.89- 2.01)	1.39	0.1642
PER 300:ZAN 10	0	0	1.07	(0.92- 1.25)	.	.	1.07	(0.92- 1.25)	.	.	.	.
PER 600:PLA	3	0.96	0.97	(0.90- 1.05)	0.97	(0.90- 1.06)	0.88	(0.59- 1.30)	1.11	(0.74- 1.66)	0.50	0.6158
PER 600:ZAN 10	0	0	1.10	(0.95- 1.28)	.	.	1.10	(0.95- 1.28)	.	.	.	.
ZAN 10:PLA	6	1.00	0.88	(0.78- 1.00)	0.88	(0.78- 1.00)	.	.	.	.	.	.

© 2021 Liu JW et al. *JAMA Network Open.*

Nausea

Random effects model
comparison - Treatment comparison
k - Number of studies providing direct evidence
prop - Direct evidence proportion
nma - Estimated treatment effect (HR) in network meta-analysis
direct - Estimated treatment effect (HR) derived from direct evidence
indir. - Estimated treatment effect (HR) derived from indirect evidence
RoR - Ratio of Ratios (direct versus indirect)
z - z-value of test for disagreement (direct versus indirect)
p-value - p-value of test for disagreement (direct versus indirect)

comparison	k	prop	nma	95%-CI	direct	95%-CI	indir.	95%-CI	RoR	95%-CI	z	p-value
BAL 40/80:OSE 150	0	0	0.40	(0.24- 0.67)	.	.	0.40	(0.24- 0.67)	.	.	.	.
BAL 40/80:OSE 75	2	0.92	0.47	(0.30- 0.72)	0.53	(0.33- 0.83)	0.12	(0.03- 0.55)	4.28	(0.89- 20.56)	1.82	0.0694
BAL 40/80:PER 300	0	0	0.83	(0.36- 1.95)	.	.	0.83	(0.36- 1.95)	.	.	.	.
BAL 40/80:PER 600	0	0	0.74	(0.32- 1.70)	.	.	0.74	(0.32- 1.70)	.	.	.	.
BAL 40/80:PLA	2	0.81	0.85	(0.54- 1.34)	0.74	(0.44- 1.22)	1.57	(0.56- 4.42)	0.47	(0.15- 1.49)	-1.28	0.1995
BAL 40/80:ZAN 10	0	0	1.55	(0.64- 3.77)	.	.	1.55	(0.64- 3.77)	.	.	.	.
OSE 150:OSE 75	2	0.92	1.17	(0.86- 1.58)	1.07	(0.78- 1.46)	3.14	(1.10- 8.97)	0.34	(0.11- 1.01)	-1.93	0.0531
OSE 150:PER 300	0	0	2.08	(0.93- 4.64)	.	.	2.08	(0.93- 4.64)	.	.	.	.
OSE 150:PER 600	0	0	1.84	(0.84- 4.04)	.	.	1.84	(0.84- 4.04)	.	.	.	.
**OSE 150:PLA**	**2**	**0.65**	**2.13**	**(1.49- 3.04)**	**2.78**	**(1.78- 4.34)**	**1.30**	**(0.71- 2.38)**	**2.13**	**(1.01- 4.51)**	**1.98**	**0.0480**
OSE 150:ZAN 10	0	0	3.88	(1.68- 8.98)	.	.	3.88	(1.68- 8.98)	.	.	.	.
OSE 75:PER 300	0	0	1.78	(0.83- 3.84)	.	.	1.78	(0.83- 3.84)	.	.	.	.
OSE 75:PER 600	1	0.06	1.58	(0.75- 3.35)	3.00	(0.13- 71.76)	1.52	(0.70- 3.29)	1.97	(0.08- 51.72)	0.41	0.6839
OSE 75:PLA	8	0.99	1.82	(1.38- 2.41)	1.81	(1.37- 2.39)	3.07	(0.29- 32.70)	0.59	(0.05- 6.38)	-0.43	0.6639
OSE 75:ZAN 10	0	0	3.33	(1.48- 7.46)	.	.	3.33	(1.48- 7.46)	.	.	.	.
PER 300:PER 600	1	0.42	0.89	(0.37- 2.13)	0.50	(0.13- 1.94)	1.34	(0.42- 4.24)	0.37	(0.06- 2.22)	-1.08	0.2784
PER 300:PLA	2	0.87	1.02	(0.50- 2.10)	1.31	(0.60- 2.82)	0.21	(0.03- 1.48)	6.31	(0.76- 52.36)	1.71	0.0878
PER 300:ZAN 10	0	0	1.87	(0.66- 5.30)	.	.	1.87	(0.66- 5.30)	.	.	.	.
PER 600:PLA	2	0.80	1.15	(0.57- 2.33)	1.08	(0.49- 2.37)	1.50	(0.31- 7.22)	0.72	(0.12- 4.18)	-0.36	0.7152
PER 600:ZAN 10	0	0	2.10	(0.75- 5.92)	.	.	2.10	(0.75- 5.92)	.	.	.	.
ZAN 10:PLA	3	1.00	0.55	(0.26- 1.17)	0.55	(0.26- 1.17)	.	.	.	.	.	.

© 2021 Liu JW et al. *JAMA Network Open.*

Vomiting

Random effects model
comparison - Treatment comparison
k - Number of studies providing direct evidence
prop - Direct evidence proportion
nma - Estimated treatment effect (HR) in network meta-analysis
direct - Estimated treatment effect (HR) derived from direct evidence
indir. - Estimated treatment effect (HR) derived from indirect evidence
RoR - Ratio of Ratios (direct versus indirect)
z - z-value of test for disagreement (direct versus indirect)
p-value - p-value of test for disagreement (direct versus indirect)

comparison	k	prop	nma	95%-CI	direct	95%-CI	indir.	95%-CI	RoR	95%-CI	z	p-value
BAL 40/80:OSE 150	0	0	0.58	(0.18- 1.87)	.	.	0.58	(0.18- 1.87)	.	.	.	.
BAL 40/80:OSE 75	1	0.89	0.69	(0.23- 2.11)	0.70	(0.22- 2.28)	0.64	(0.02- 18.33)	1.10	(0.03- 38.40)	0.05	0.9600
BAL 40/80:PER 300	0	0	2.15	(0.47- 9.95)	.	.	2.15	(0.47- 9.95)	.	.	.	.
BAL 40/80:PLA	1	0.48	1.31	(0.42- 4.03)	1.27	(0.25- 6.49)	1.34	(0.28- 6.37)	0.94	(0.10- 9.03)	-0.05	0.9600
BAL 40/80:ZAN 10	0	0	1.18	(0.26- 5.46)	.	.	1.18	(0.26- 5.46)	.	.	.	.
**OSE 150:OSE 75**	**2**	**0.92**	**1.19**	**(0.84- 1.68)**	**1.05**	**(0.73- 1.50)**	**4.91**	**(1.48- 16.30)**	**0.21**	**(0.06- 0.74)**	**-2.42**	**0.0154**
OSE 150:PER 300	0	0	3.69	(1.22- 11.17)	.	.	3.69	(1.22- 11.17)	.	.	.	.
**OSE 150:PLA**	**2**	**0.47**	**2.24**	**(1.51- 3.32)**	**3.70**	**(2.08- 6.57)**	**1.43**	**(0.83- 2.46)**	**2.59**	**(1.17- 5.72)**	**2.36**	**0.0184**
OSE 150:ZAN 10	0	0	2.03	(0.67- 6.12)	.	.	2.03	(0.67- 6.12)	.	.	.	.
OSE 75:PER 300	0	0	3.10	(1.07- 8.99)	.	.	3.10	(1.07- 8.99)	.	.	.	.
OSE 75:PLA	7	1.00	1.88	(1.47- 2.41)	1.88	(1.47- 2.41)	.	.	.	.	.	.
OSE 75:ZAN 10	0	0	1.71	(0.59- 4.93)	.	.	1.71	(0.59- 4.93)	.	.	.	.
PER 300:PLA	1	1.00	0.61	(0.22- 1.71)	0.61	(0.22- 1.71)	.	.	.	.	.	.
PER 300:ZAN 10	0	0	0.55	(0.13- 2.37)	.	.	0.55	(0.13- 2.37)	.	.	.	.
ZAN 10:PLA	1	1.00	1.10	(0.39- 3.09)	1.10	(0.39- 3.09)	.	.	.	.	.	.

© 2021 Liu JW et al. *JAMA Network Open.*

eTable 3. Sensitivity Analysis

Studies at serious risk of bias (ROB) of overall domains and blinding domain were excluded.

For the TTAS outcome, 1 trial (Lin, 2006) was excluded in both domains.
For the nausea outcome, 1 trial (Lin, 2006) was excluded in the overall domain; (Nakamura, 2017) and (Lin, 2006) were excluded in the domain of blinding.
No trial had a high risk of bias for the vomiting outcome.

Time to symptom alleviation (TTAS)

	Base data			**Overall / Blinding domains**		
Comparison	No.	Prop	RR (95% CI)	No.	Prop	RR (95% CI)
BAL 40/80 vs OSE 150	0	0	1.06 (0.91-1.24)	0	0	1.06 (0.91-1.24)
BAL 40/80 vs OSE 75	2	0.85	1.07 (0.98-1.16)	2	0.85	1.06 (0.98- 1.16)
BAL 40/80 vs PER 300	0	0	1.06 (0.86-1.30)	0	0	1.06 (0.86- 1.31)
BAL 40/80 vs PER 600	0	0	1.15 (0.88-1.50)	0	0	1.16 (0.89- 1.51)
BAL 40/80 vs PLA	3	0.84	0.79 (0.73-0.86)	3	0.84	0.80 (0.73- 0.87)
BAL 40/80 vs ZAN 10	0	0	1.19 (1.01-1.40)	0	0	1.19 (1.01- 1.41)
OSE 150 vs OSE 75	2	0.74	1.00 (0.87-1.16)	2	0.74	1.00 (0.87- 1.15)
OSE 150 vs PER 300	0	0	0.99 (0.78-1.26)	0	0	1.00 (0.79- 1.27)
OSE 150 vs PER 600	0	0	1.08 (0.81-1.44)	0	0	1.09 (0.82- 1.45)
OSE 150 vs PLA	2	0.78	0.75 (0.65-0.86)	2	0.79	0.75 (0.65- 0.86)
OSE 150 vs ZAN 10	0	0	1.12 (0.91-1.36)	0	0	1.12 (0.92- 1.37)
OSE 75 vs PER 300	0	0	0.99 (0.81-1.21)	0	0	1.00 (0.81- 1.22)
OSE 75 vs PER 600	0	0	1.08 (0.83-1.40)	0	0	1.09 (0.84- 1.41)
**OSE 75 vs PLA**	9	0.98	0.74 (0.70-0.79)	**8***	0.98	0.75 (0.70- 0.80)
OSE 75 vs ZAN 10	0	0	1.11 (0.95-1.30)	0	0	1.12 (0.96- 1.31)
PER 300 vs PER 600	1	0.84	1.09 (0.85-1.40)	1	0.84	1.09 (0.85- 1.40)
PER 300 vs PLA	2	1.00	0.75 (0.62-0.91)	2	1.00	0.75 (0.62- 0.91)
PER 300 vs ZAN 10	0	0	1.12 (0.88-1.43)	0	0	1.12 (0.88- 1.43)
PER 600 vs PLA	1	0.84	0.69 (0.54-0.88)	1	0.84	0.69 (0.54- 0.88)
PER 600 vs ZAN 10	0	0	1.03 (0.77-1.37)	0	0	1.03 (0.77- 1.37)
PLA vs ZAN 10	3	1.00	1.50 (1.30-1.73)	3	1.00	1.50 (1.30- 1.73)

No: numbers of studies included. Prop: Direct evidence proportion. RR: risk ratio. CI: confident interval.
*Excluding Lin, 2006 in both domains.

© 2021 Liu JW et al. *JAMA Network Open.*

Nausea

	Base data			**Overall domains**			**Blinding domains**		
Comparison	No.	Prop	RR (95% CI)	No.	Prop	RR (95% CI)	No.	Prop	RR (95% CI)
BAL 40/80 vs OSE 150	0	0	0.40 (0.24-0.67)	0	0	0.40 (0.24-0.67)	0	0	0.40 (0.24-0.67)
BAL 40/80 vs OSE 75	2	0.92	0.47 (0.30-0.72)	2	0.92	0.47 (0.30-0.73)	2	0.92	0.47 (0.30-0.73)
BAL 40/80 vs PER 300	0	0	0.83 (0.36-1.95)	0	0	0.83 (0.35-1.94)	0	0	0.82 (0.35-1.92)
BAL 40/80 vs PER 600	0	0	0.74 (0.32-1.70)	0	0	0.73 (0.32-1.69)	0	0	0.71 (0.30-1.66)
BAL 40/80 vs PLA	2	0.81	0.85 (0.54-1.34)	2	0.81	0.85 (0.54-1.34)	2	0.81	0.85 (0.54-1.33)
BAL 40/80 vs ZAN 10	0	0	1.55 (0.64-3.77)	0	0	1.55 (0.64-3.75)	0	0	1.54 (0.64-3.74)
OSE 150 vs OSE 75	2	0.92	1.17 (0.86-1.58)	2	0.92	1.17 (0.87-1.58)	2	0.92	1.17 (0.87-1.58)
OSE 150 vs PER 300	0	0	2.08 (0.93-4.64)	0	0	2.07 (0.93-4.61)	0	0	2.04 (0.92-4.56)
OSE 150 vs PER 600	0	0	1.84 (0.84-4.04)	0	0	1.83 (0.84-4.02)	0	0	1.77 (0.79-3.95)
OSE 150 vs PLA	2	0.65	2.13 (1.49-3.04)	2	0.65	2.11 (1.48-3.03)	2	0.65	2.11 (1.47-3.02)
OSE 150 vs ZAN 10	0	0	3.88 (1.68-8.98)	0	0	3.86 (1.67-8.93)	0	0	3.84 (1.66-8.89)
OSE 75 vs PER 300	0	0	1.78 (0.83-3.84)	0	0	1.77 (0.82-3.81)	0	0	1.75 (0.81-3.77)
**OSE 75 vs PER 600**	1	0.06	1.58 (0.75-3.35)	1	0.06	1.57 (0.74-3.32)	**0†**	0	1.51 (0.70-3.26)
**OSE 75 vs PLA**	8	0.99	1.82 (1.38-2.41)	**7***	0.99	1.81 (1.37-2.39)	**7†**	0.99	1.80 (1.36-2.38)
OSE 75 vs ZAN 10	0	0	3.33 (1.48-7.46)	0	0	3.30 (1.47-7.40)	0	0	3.28 (1.46-7.36)
PER 300 vs PER 600	1	0.42	0.89 (0.37-2.13)	1	0.42	0.89 (0.37-2.13)	1	0.43	0.86 (0.36-2.10)
PER 300 vs PLA	2	0.87	1.02 (0.50-2.10)	2	0.87	1.02 (0.50-2.10)	2	0.87	1.03 (0.50-2.11)
PER 300 vs ZAN 10	0	0	1.87 (0.66-5.30)	0	0	1.86 (0.66-5.30)	0	0	1.88 (0.66-5.35)
PER 600 vs PLA	2	0.80	1.15 (0.57-2.33)	2	0.80	1.15 (0.57-2.33)	2	0.84	1.19 (0.58-2.45)
PER 600 vs ZAN 10	0	0	2.10 (0.75-5.92)	0	0	2.10 (0.75-5.92)	0	0	2.18 (0.76-6.19)
PLA vs ZAN 10	3	1.00	0.55 (0.26-1.17)	3	1.00	0.55 (0.26-1.17)	3	1.00	0.55 (0.26-1.17)

No: numbers of studies included. Prop: Direct evidence proportion. RR: risk ratio. CI: confident interval.
*Excluding Lin, 2006 in overall domains.
†Excluding Nakamura, 2017 and Lin, 2006 in blinding domain.

© 2021 Liu JW et al. *JAMA Network Open.*

eTable 4. Mantel-Haenszel (MH) Method for Binary Outcomes

Outcomes		Network meta-analysis (ref = PLA)	
**Complications**	**Comparison***	**Base data RR (95% CI)**	**MH method OR (95% CI)**
	BAL 40/80	0.51 (0.32- 0.80)	0.45 (0.28- 0.71)
	OSE 150	0.65 (0.41- 1.02)	0.60 (0.36- 0.99)
	OSE 75	0.61 (0.49- 0.75)	0.56 (0.44- 0.72)
	PER 300	0.67 (0.40- 1.12)	0.64 (0.35- 1.14)
	PER 600	0.65 (0.37- 1.16)	0.58 (0.30- 1.13)
	ZAN 10	0.82 (0.72- 0.92)	0.73 (0.61- 0.87)
**Total adverse events**	**Comparison***	**Base data RR (95% CI)**	**MH method OR (95% CI)**
	BAL 40/80	0.84 (0.74- 0.96)	0.80 (0.68- 0.95)
	OSE 150	0.97 (0.60- 1.59)	0.99 (0.54- 1.79)
	OSE 75	0.96 (0.87- 1.06)	0.97 (0.85- 1.12)
	PER 300	0.95 (0.87- 1.03)	0.77 (0.53- 1.13)
	PER 600	0.97 (0.90- 1.05)	0.75 (0.53- 1.04)
	ZAN 10	0.88 (0.78- 1.00)	0.84 (0.70- 1.00)
**Nausea**	**Comparison***	**Base data RR (95% CI)**	**MH method OR (95% CI)**
	BAL 40/80	0.85 (0.54- 1.34)	0.88 (0.55- 1.40)
	OSE 150	2.13 (1.49- 3.04)	2.34 (1.57- 3.49)
	OSE 75	1.82 (1.38- 2.41)	1.92 (1.44- 2.57)
	PER 300	1.02 (0.50- 2.10)	1.03 (0.48- 2.21)
	PER 600	1.15 (0.57- 2.33)	1.18 (0.56- 2.48)
	ZAN 10	0.55 (0.26- 1.17)	0.54 (0.25- 1.17)
**Vomiting**	**Comparison***	**Base data RR (95% CI)**	**MH method OR (95% CI)**
	BAL 40/80	1.31 (0.42- 4.03)	1.42 (0.45- 4.46)
	OSE 150	2.24 (1.51- 3.32)	2.56 (1.64- 3.98)
	OSE 75	1.88 (1.47- 2.41)	2.11 (1.59- 2.81)
	PER 300	0.61 (0.22- 1.71)	0.59 (0.20- 1.75)
	ZAN 10	1.10 (0.39- 3.09)	1.11 (0.38- 3.20)

RR: risk ratio. OR: odds ratio. CI: confident interval.
*Comparison: antiviral agents comparing placebo.

© 2021 Liu JW et al. *JAMA Network Open.*

eFigure 4. Comparison-Adjusted Funnel Plot

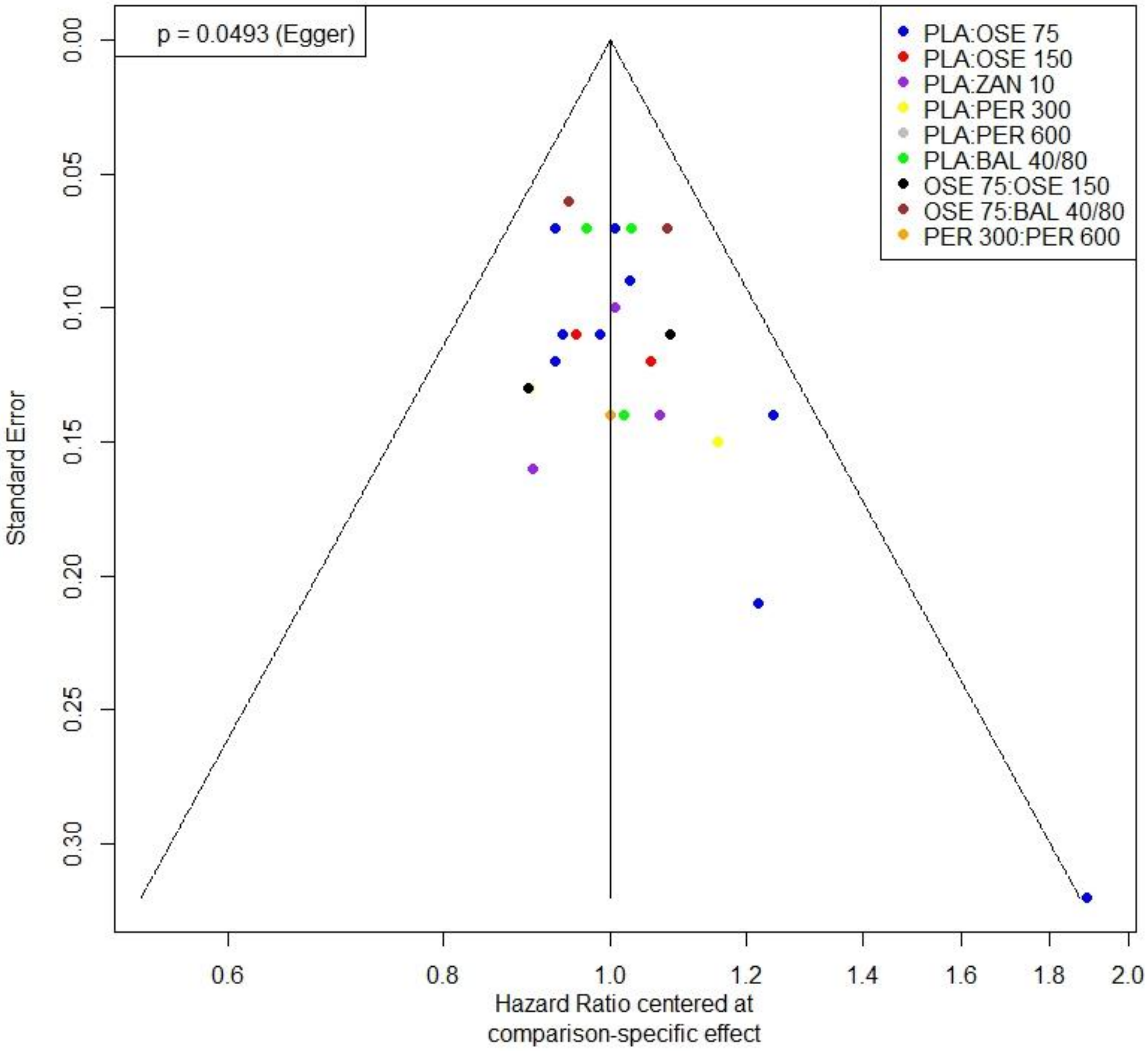

© 2021 Liu JW et al. *JAMA Network Open.*

eTable 5. Certainty of Direct Evidence Assessment

Comparison	No.	Risk of bias	Inconsistency	Indirectness	Publication bias	HR (95% CI)	**Certainty of evidence**
**TTAS**							
BAL 40/80 vs OSE 75	2	Serious[5]	Not Serious	Not Serious	Unclear[2]	1.05 (0.96-1.14)	**Moderate**
BAL 40/80 vs PLA	3	Serious[5]	Not Serious	Not Serious	Unclear[2]	0.81 (0.74-0.89)	**Moderate**
OSE 150 vs OSE 75	2	Not Serious	Not Serious	Not Serious	Unclear[2]	1.02 (0.86-1.20)	**High**
OSE 150 vs PLA	2	Not Serious	Not Serious	Not Serious	Unclear[2]	0.74 (0.63-0.86)	**High**
OSE 75 vs PLA	9	Serious[3,4,5]	Not Serious	Not Serious	Unclear[2]	0.74 (0.69-0.79)	**Moderate**
PER 300 vs PER 600	1	Not Serious	NA[1]	Not Serious	Unclear[2]	1.01 (0.77-1.33)	**High**
PER 300 vs PLA	2	Not Serious	Not Serious	Not Serious	Unclear[2]	0.75 (0.62-0.91)	**High**
PER 600 vs PLA	1	Not Serious	NA[1]	Not Serious	Unclear[2]	0.64 (0.48-0.84)	**High**
PLA vs ZAN 10	3	Not Serious	Not Serious	Not Serious	Unclear[2]	1.50 (1.30-1.73)	**High**
**Complications**							
BAL 40/80 vs OSE 75	2	Serious[5]	Not Serious	Not Serious	Unclear[2]	0.91 (0.53-1.57)	**Moderate**
BAL 40/80 vs PLA	3	Serious[5]	Not Serious	Not Serious	Unclear[2]	0.44 (0.27-0.72)	**Moderate**
OSE 150 vs OSE 75	2	Not Serious	Not Serious	Not Serious	Unclear[2]	0.94 (0.56-1.58)	**High**
OSE 150 vs PLA	2	Not Serious	Not Serious	Not Serious	Unclear[2]	0.69 (0.42-1.12)	**High**
OSE 75 vs PER 600	2	Serious[3]	Not Serious	Not Serious	Unclear[2]	1.62 (0.53-4.95)	**Moderate**
OSE 75 vs PLA	9	Serious[3,4,5]	Not Serious	Not Serious	Unclear[2]	0.61 (0.49-0.75)	**Moderate**
PER 300 vs PER 600	2	Not Serious	Not Serious	Not Serious	Unclear[2]	1.60 (0.38-6.66)	**High**
PER 300 vs PLA	2	Not Serious	Not Serious	Not Serious	Unclear[2]	0.65 (0.38-1.11)	**High**
PER 600 vs PLA	3	Not Serious	Not Serious	Not Serious	Unclear[2]	0.82 (0.41-1.64)	**High**
ZAN 10 vs PLA	6	Not Serious	Not Serious	Not Serious	Unclear[2]	0.82 (0.72-0.92)	**High**
**Any adverse event**							
BAL 40/80 vs OSE 75	2	Serious[5]	Not Serious	Not Serious	Unclear[2]	0.87 (0.76-1.00)	**Moderate**
BAL 40/80 vs PLA	3	Serious[5]	Not Serious	Not Serious	Unclear[2]	0.85 (0.74-0.97)	**Moderate**
OSE 150 vs OSE 75	1	Not Serious	NA[1]	Not Serious	Unclear[2]	1.01 (0.62-1.64)	**High**
OSE 75 vs PER 600	2	Serious[3]	Not Serious	Not Serious	Unclear[2]	1.61 (0.83-3.12)	**Moderate**
OSE 75 vs PLA	8	Serious[3,4,5]	Not Serious	Not Serious	Unclear[2]	0.95 (0.86-1.05)	**Moderate**
PER 300 vs PER 600	2	Not Serious	Not Serious	Not Serious	Unclear[2]	0.96 (0.88-1.06)	**High**
PER 300 vs PLA	2	Not Serious	Not Serious	Not Serious	Unclear[2]	0.96 (0.88-1.05)	**High**
PER 600 vs PLA	3	Not Serious	Not Serious	Not Serious	Unclear[2]	0.97 (0.90-1.06)	**High**

© 2021 Liu JW et al. *JAMA Network Open.*

ZAN 10 vs PLA	6	Not Serious	Not Serious	Not Serious	Unclear[2]	0.88 (0.78-1.00)	**High**
**Nausea**							
BAL 40/80 vs OSE 75	2	Serious[5]	Not Serious	Not Serious	Unclear[2]	0.53 (0.33-0.83)	**Moderate**
BAL 40/80 vs PLA	2	Serious[5]	Not Serious	Not Serious	Unclear[2]	0.74 (0.44-1.22)	**Moderate**
OSE 150 vs OSE 75	2	Not Serious	Not Serious	Not Serious	Unclear[2]	1.07 (0.78-1.46)	**High**
OSE 150 vs PLA	2	Not Serious	Not Serious	Not Serious	Unclear[2]	2.78 (1.78-4.34)	**High**
OSE 75 vs PER 600	1	Serious[3]	NA[1]	Not Serious	Unclear[2]	3.00 (0.13-71.76)	**Moderate**
OSE 75 vs PLA	8	Serious[3,4,5]	Not Serious	Not Serious	Unclear[2]	1.81 (1.37-2.39)	**Moderate**
PER 300 vs PER 600	1	Not Serious	NA[1]	Not Serious	Unclear[2]	0.50 (0.13-1.94)	**High**
PER 300 vs PLA	2	Not Serious	Not Serious	Not Serious	Unclear[2]	1.31 (0.60-2.82)	**High**
PER 600 vs PLA	2	Not Serious	Not Serious	Not Serious	Unclear[2]	1.08 (0.49-2.37)	**High**
ZAN 10 vs PLA	3	Not Serious	Not Serious	Not Serious	Unclear[2]	0.55 (0.26-1.17)	**High**
**Vomiting**							
BAL 40/80 vs OSE 75	1	Not Serious	NA[1]	Not Serious	Unclear[2]	0.70 (0.22-2.28)	**High**
BAL 40/80 vs PLA	1	Not Serious	NA[1]	Not Serious	Unclear[2]	1.27 (0.25-6.49)	**High**
OSE 150 vs OSE 75	2	Not Serious	Not Serious	Not Serious	Unclear[2]	1.05 (0.73-1.50)	**High**
OSE 150 vs PLA	2	Not Serious	Not Serious	Not Serious	Unclear[2]	3.70 (2.08-6.57)	**High**
OSE 75 vs PLA	7	Not Serious	Not Serious	Not Serious	Unclear[2]	1.88 (1.47-2.41)	**High**
PER 300 vs PLA	1	Not Serious	NA[1]	Not Serious	Unclear[2]	0.61 (0.22-1.71)	**High**
ZAN 10 vs PLA	1	Not Serious	NA[1]	Not Serious	Unclear[2]	1.10 (0.39-3.09)	**High**

1. Unable to assess because there are <2 studies available with non-zero events in both arms.
2. As per protocol, the funnel plot or Egger's test was not performed because of insufficient information (<10 studies).
3. Studies failed to blind patients and investigators that might lead to performance bias.
4. Studies had inadequate randomization and assignments that might be anticipated.
5. Studies had incomplete outcome data given that drop-outs and people lost to follow-ups could have clinically relevant impact on the intervention effect estimates.

© 2021 Liu JW et al. *JAMA Network Open.*

eTable 6. Certainty of Network Evidence Assessment

Comparison	Direct Evidence		Indirect Evidence		Network Meta-Analysis	
	HR (95% CI)	Certainty of Evidence	HR (95% CI)	Certainty of Evidence	HR (95% CI)	Certainty of Evidence
**TTAS (ITTI analysis) (n = 15)**						
BAL 40/80 vs OSE 150	-	-	1.06 (0.91-1.24)	**Moderate**[4]	1.06 (0.91-1.24)	**Low**[2]
BAL 40/80 vs OSE 75	1.05 (0.96-1.14)	Moderate	1.20 (0.97-1.48)	**Moderate**[4]	1.07 (0.98-1.16)	**Low**[2]
BAL 40/80 vs PER 300	-	-	1.06 (0.86-1.30)	**Moderate**[4]	1.06 (0.86-1.30)	**Low**[2]
BAL 40/80 vs PER 600	-	-	1.15 (0.88-1.50)	**Low**[4,5]	1.15 (0.88-1.50)	**Very low**[2]
BAL 40/80 vs PLA	0.81 (0.74-0.89)	Moderate	0.71 (0.58-0.88)	**Moderate**[4]	0.79 (0.73-0.86)	**Moderate**
BAL 40/80 vs ZAN 10	-	-	1.19 (1.01-1.40)	**Low**[4,5]	1.19 (1.01-1.40)	**Low**
OSE 150 vs OSE 75	1.02 (0.86-1.20)	High	0.96 (0.73-1.27)	Not needed[1]	1.00 (0.87-1.16)	**Moderate**[2]
OSE 150 vs PER 300	-	-	0.99 (0.78-1.26)	High[3]	0.99 (0.78-1.26)	**Moderate**[2]
OSE 150 vs PER 600	-	-	1.08 (0.81-1.44)	**Moderate**[3,5]	1.08 (0.81-1.44)	**Low**[2]
OSE 150 vs PLA	0.74 (0.63-0.86)	High	0.78 (0.58-1.06)	Not needed[1]	0.75 (0.65-0.86)	**High**
OSE 150 vs ZAN 10	-	-	1.12 (0.91-1.36)	**Moderate**[3,5]	1.12 (0.91-1.36)	**Low**[2]
OSE 75 vs PER 300	-	-	0.99 (0.81-1.21)	**Moderate**[4]	0.99 (0.81-1.21)	**Low**[2]
OSE 75 vs PER 600	-	-	1.08 (0.83-1.40)	**Low**[4,5]	1.08 (0.83-1.40)	**Very low**[2]
OSE 75 vs PLA	0.74 (0.69-0.79)	Moderate	0.99 (0.62-1.56)	**Moderate**[4]	0.74 (0.70-0.79)	**Moderate**
OSE 75 vs ZAN 10	-	-	1.11 (0.95-1.30)	**Moderate**[4]	1.11 (0.95-1.30)	**Low**[2]
PER 300 vs PER 600	1.01 (0.77-1.33)	High	1.61 (0.87-2.99)	Not needed[1]	1.09 (0.85-1.40)	**Moderate**[2]
PER 300 vs PLA	0.75 (0.62-0.91)	High	-	-	0.75 (0.62-0.91)	**High**
PER 300 vs ZAN 10	-	-	1.12 (0.88-1.43)	**High**[3]	1.12 (0.88-1.43)	**Moderate**[2]
PER 600 vs PLA	0.64 (0.48-0.84)	High	1.02 (0.55-1.89)	Not needed[1]	0.69 (0.54-0.88)	**High**
PER 600 vs ZAN 10	-	-	1.03 (0.77-1.37)	**Moderate**[3,5]	1.03 (0.77-1.37)	**Low**[2]
PLA vs ZAN 10	1.50 (1.30-1.73)	High	-	-	1.50 (1.30-1.73)	**High**
**Complications (ITTI analysis) (n = 21)**						
BAL 40/80 vs OSE 150	-	-	0.78 (0.41-1.46)	**Moderate**[4]	0.78 (0.41-1.46)	**Low**[2]
BAL 40/80 vs OSE 75	0.91 (0.53-1.57)	Moderate	0.63 (0.25-1.58)	**Moderate**[4]	0.83 (0.52-1.32)	**Low**[2]
BAL 40/80 vs PER 300	-	-	0.75 (0.38-1.49)	**Moderate**[4]	0.75 (0.38-1.49)	**Low**[2]
BAL 40/80 vs PER 600	-	-	0.77 (0.37-1.59)	**Low**[4,5]	0.77 (0.37-1.59)	**Very Low**[2]
BAL 40/80 vs PLA	0.44 (0.27-0.72)	Moderate	1.17 (0.35-3.94)	**Moderate**[4]	0.51 (0.32-0.80)	**Moderate**
BAL 40/80 vs ZAN 10	-	-	0.62 (0.39-0.99)	**Moderate**[4]	0.62 (0.39-0.99)	**Moderate**

© 2021 Liu JW et al. *JAMA Network Open.*

OSE 150 vs OSE 75	0.94 (0.56-1.58)	High	1.71 (0.64-4.55)	Not needed[1]	1.07 (0.67-1.69)	**Moderate**[2]
OSE 150 vs PER 300	-	-	0.97 (0.49-1.92)	**High**[3]	0.97 (0.49-1.92)	**Moderate**[2]
OSE 150 vs PER 600	-	-	0.99 (0.48-2.04)	**Moderate**[3,5]	0.99 (0.48-2.04)	**Low**[2]
OSE 150 vs PLA	0.69 (0.42-1.12)	High	0.45 (0.13-1.53)	Not needed[1]	0.65 (0.41-1.02)	**Moderate**[2]
OSE 150 vs ZAN 10	-	-	0.80 (0.50-1.27)	**High**[3]	0.80 (0.50-1.27)	**Moderate**[2]
OSE 75 vs PER 300	-	-	0.91 (0.52-1.57)	**Moderate**[4]	0.91 (0.52-1.57)	**Low**[2]
OSE 75 vs PER 600	1.62 (0.53-4.95)	Moderate	0.75 (0.38-1.51)	**Low**[4,5]	0.93 (0.52-1.68)	**Very Low**[2]
OSE 75 vs PLA	0.61 (0.49-0.75)	Moderate	0.74 (0.21-2.62)	**Moderate**[4]	0.61 (0.49-0.75)	**Moderate**
OSE 75 vs ZAN 10	-	-	0.75 (0.59-0.95)	**Moderate**[4]	0.75 (0.59-0.95)	**Moderate**
PER 300 vs PER 600	1.60 (0.38-6.66)	High	0.88 (0.38-2.03)	Not needed[1]	1.03 (0.50-2.11)	**Moderate**[2]
PER 300 vs PLA	0.65 (0.38-1.11)	High	0.95 (0.15-6.00)	Not needed[1]	0.67 (0.40-1.12)	**Moderate**[2]
PER 300 vs ZAN 10	-	-	0.82 (0.49-1.39)	**High**[3]	0.82 (0.49-1.39)	**Moderate**[2]
PER 600 vs PLA	0.82 (0.41-1.64)	High	0.40 (0.15-1.10)	Not needed[1]	0.65 (0.37-1.16)	**Moderate**[2]
PER 600 vs ZAN 10	-	-	0.80 (0.45-1.43)	**Moderate**[3]	0.80 (0.45-1.43)	**Low**[2]
ZAN 10 vs PLA	0.82 (0.72-0.92)	High	-	-	0.82 (0.72-0.92)	High
**Any adverse event (As-treated) (n = 21)**						
BAL 40/80 vs OSE 150	-	-	0.86 (0.53-1.42)	**Low**[4,5]	0.86 (0.53-1.42)	**Very Low**[2]
BAL 40/80 vs OSE 75	0.87 (0.76-1.00)	Moderate	0.90 (0.64-1.27)	**Moderate**[4]	0.87 (0.77-0.99)	**Moderate**
BAL 40/80 vs PER 300	-	-	0.89 (0.77-1.04)	**Moderate**[4]	0.89 (0.77-1.04)	**Low**[2]
BAL 40/80 vs PER 600	-	-	0.87 (0.75-1.01)	**Low**[4,5]	0.87 (0.75-1.01)	**Very Low**[2]
BAL 40/80 vs PLA	0.85 (0.74-0.97)	Moderate	0.81 (0.57-1.17)	**Moderate**[4]	0.84 (0.74-0.96)	**Moderate**
BAL 40/80 vs ZAN 10	-	-	0.96 (0.80-1.14)	**Moderate**[4]	0.96 (0.80-1.14)	**Low**[2]
OSE 150 vs OSE 75	1.01 (0.62-1.64)	High	-	-	1.01 (0.62-1.64)	**Moderate**[2]
OSE 150 vs PER 300	-	-	1.03 (0.63-1.70)	**Low**[4,5]	1.03 (0.63-1.70)	**Very Low**[2]
OSE 150 vs PER 600	-	-	1.01 (0.61-1.65)	**Low**[4,5]	1.01 (0.61-1.65)	**Very Low**[2]
OSE 150 vs PLA	-	-	0.97 (0.60-1.59)	**Low**[4,5]	0.97 (0.60-1.59)	**Very Low**[2]
OSE 150 vs ZAN 10	-	-	1.11 (0.67-1.84)	**Low**[4,5]	1.11 (0.67-1.84)	**Very Low**[2]
OSE 75 vs PER 300	-	-	1.02 (0.89-1.16)	**Moderate**[4]	1.02 (0.89-1.16)	**Low**[2]
OSE 75 vs PER 600	1.61 (0.83-3.12)	Moderate	0.98 (0.86-1.11)	**Low**[4,5]	0.99 (0.88-1.13)	**Low**
OSE 75 vs PLA	0.95 (0.86-1.05)	Moderate	1.52 (0.83-2.78)	**Moderate**[4]	0.96 (0.87-1.06)	**Low**[2]
OSE 75 vs ZAN 10	-	-	1.09 (0.93-1.28)	**Moderate**[4]	1.09 (0.93-1.28)	**Low**[2]
PER 300 vs PER 600	0.96 (0.88-1.06)	High	1.12 (0.80-1.57)	Not needed[1]	0.98 (0.89-1.07)	**Moderate**[2]
PER 300 vs PLA	0.96 (0.88-1.05)	High	0.72 (0.48-1.07)	Not needed[1]	0.95 (0.87-1.03)	**Moderate**[2]

© 2021 Liu JW et al. *JAMA Network Open.*

PER 300 vs ZAN 10	-	-	1.07 (0.92-1.25)	**High**[3]	1.07 (0.92-1.25)	**Moderate**[2]
PER 600 vs PLA	0.97 (0.90-1.06)	High	0.88 (0.59-1.30)	Not needed[1]	0.97 (0.90-1.05)	**Moderate**[2]
PER 600 vs ZAN 10	-	-	1.10 (0.95-1.28)	**Moderate**[3,5]	1.10 (0.95-1.28)	**Low**[2]
ZAN 10 vs PLA	0.88 (0.78-1.00)	High	-	-	0.88 (0.78-1.00)	**Moderate**[2]
**Nausea (As-treated) (n = 15)**						
BAL 40/80 vs OSE 150	-	-	0.40 (0.24-0.67)	**Moderate**[4]	0.40 (0.24-0.67)	**Moderate**
BAL 40/80 vs OSE 75	0.53 (0.33-0.83)	Moderate	0.12 (0.03-0.55)	**Moderate**[4]	0.47 (0.30-0.72)	**Moderate**
BAL 40/80 vs PER 300	-	-	0.83 (0.36-1.95)	**Moderate**[4]	0.83 (0.36-1.95)	**Low**[2]
BAL 40/80 vs PER 600	-	-	0.74 (0.32-1.70)	**Low**[4,5]	0.74 (0.32-1.70)	**Very Low**[2]
BAL 40/80 vs PLA	0.74 (0.44-1.22)	Moderate	1.57 (0.56-4.42)	**Moderate**[4]	0.85 (0.54-1.34)	**Low**[2]
BAL 40/80 vs ZAN 10	-	-	1.55 (0.64-3.77)	**Moderate**[4]	1.55 (0.64-3.77)	**Low**[2]
OSE 150 vs OSE 75	1.07 (0.78-1.46)	High	3.14 (1.10-8.97)	Not needed[1]	1.17 (0.86-1.58)	**Moderate**[2]
OSE 150 vs PER 300	-	-	2.08 (0.93-4.64)	**High**[3]	2.08 (0.93-4.64)	**Moderate**[2]
OSE 150 vs PER 600	-	-	1.84 (0.84-4.04)	**Moderate**[3,5]	1.84 (0.84-4.04)	**Low**[2]
OSE 150 vs PLA	2.78 (1.78-4.34)	High	1.30 (0.71-2.38)	Not needed[1]	2.13 (1.49-3.04)	**High**
OSE 150 vs ZAN 10	-	-	3.88 (1.68-8.98)	**High**[3]	3.88 (1.68-8.98)	**High**
OSE 75 vs PER 300	-	-	1.78 (0.83-3.84)	**Moderate**[4]	1.78 (0.83-3.84)	**Low**[2]
OSE 75 vs PER 600	3.00 (0.13-71.76)	Moderate	1.52 (0.70-3.29)	**Low**[4,5]	1.58 (0.75-3.35)	**Very Low**[2]
OSE 75 vs PLA	1.81 (1.37-2.39)	Moderate	3.07 (0.29-32.70)	**Moderate**[4]	1.82 (1.38-2.41)	**Moderate**
OSE 75 vs ZAN 10	-	-	3.33 (1.48-7.46)	**Low**[4,5]	3.33 (1.48-7.46)	**Low**
PER 300 vs PER 600	0.50 (0.13-1.94)	High	1.34 (0.42-4.24)	**Moderate**[3,5]	0.89 (0.37-2.13)	**Low**[2]
PER 300 vs PLA	1.31 (0.60-2.82)	High	0.21 (0.03-1.48)	Not needed[1]	1.02 (0.50-2.10)	**Moderate**[2]
PER 300 vs ZAN 10	-	-	1.87 (0.66-5.30)	**High**[3]	1.87 (0.66-5.30)	**Moderate**[2]
PER 600 vs PLA	1.08 (0.49-2.37)	High	1.50 (0.31-7.22)	Not needed[1]	1.15 (0.57-2.33)	**Moderate**[2]
PER 600 vs ZAN 10	-	-	2.10 (0.75-5.92)	**Moderate**[5]	2.10 (0.75-5.92)	**Low**[2]
ZAN 10 vs PLA	0.55 (0.26-1.17)	High	-	-	0.55 (0.26-1.17)	**Moderate**[2]
**Vomiting (As-treated) (n = 9)**						
BAL 40/80 vs OSE 150	-	-	0.58 (0.18-1.87)	**High**[3]	0.58 (0.18-1.87)	**Moderate**[2]
BAL 40/80 vs OSE 75	0.70 (0.22-2.28)	High	0.64 (0.02-18.33)	Not needed[1]	0.69 (0.23-2.11)	**Moderate**[2]
BAL 40/80 vs PER 300	-	-	2.15 (0.47-9.95)	**High**[3]	2.15 (0.47-9.95)	**Moderate**[2]
BAL 40/80 vs PLA	1.27 (0.25-6.49)	High	1.34 (0.28-6.37)	**High**[3]	1.31 (0.42-4.03)	**Moderate**[2]
BAL 40/80 vs ZAN 10	-	-	1.18 (0.26-5.46)	**High**[3]	1.18 (0.26-5.46)	**Moderate**[2]

© 2021 Liu JW et al. *JAMA Network Open.*

OSE 150 vs OSE 75	1.05 (0.73-1.50)	High	4.91 (1.48-16.30)	**High**[3]	1.19 (0.84-1.68)	**Moderate**[2]
OSE 150 vs PER 300	-	-	3.69 (1.22-11.17)	**High**[3]	3.69 (1.22-11.17)	**High**
OSE 150 vs PLA	3.70 (2.08-6.57)	High	1.43 (0.83-2.46)	**High**[3]	2.24 (1.51-3.32)	**Moderate**[6]
OSE 150 vs ZAN 10	-	-	2.03 (0.67-6.12)	**High**[3]	2.03 (0.67-6.12)	**Moderate**[2]
OSE 75 vs PER 300	-	-	3.10 (1.07-8.99)	**High**[3]	3.10 (1.07-8.99)	**Moderate**[2]
OSE 75 vs PLA	1.88 (1.47-2.41)	High	-	-	1.88 (1.47-2.41)	**High**
OSE 75 vs ZAN 10	-	-	1.71 (0.59-4.93)	**High**[3]	1.71 (0.59-4.93)	**Moderate**[2]
PER 300 vs PLA	0.61 (0.22-1.71)	High	-	-	0.61 (0.22-1.71)	**Moderate**[2]
PER 300 vs ZAN 10	-	-	0.55 (0.13-2.37)	**High**[3]	0.55 (0.13-2.37)	**Moderate**[2]
ZAN 10 vs PLA	1.10 (0.39-3.09)	High	-	-	1.10 (0.39-3.09)	**Moderate**[2]

1. There is no need to rate the indirect evidence since the certainty of the direct evidence is high and the contribution of the direct evidence to the network estimate is much greater than that of the indirect evidence.
2. Imprecise (wide 95% CI).
3. The confidence ratings for both direct comparisons are high.
4. The lower confidence rating of the two direct comparisons is moderate.
5. Probable intransitivity due to different severity of illness (outpatients or hospitalized patients).
6. Incoherence between direct and indirect evidence (dominant estimate not similar to network estimate).

© 2021 Liu JW et al. *JAMA Network Open.*
